# Expression of the *lux* genes in *Streptococcus pneumoniae* modulates pilus expression and virulence

**DOI:** 10.1371/journal.pone.0189426

**Published:** 2018-01-17

**Authors:** Jenny A. Herbert, Andrea M. Mitchell, Ryan Ritchie, Jiangtao Ma, Kirsty Ross-Hutchinson, Timothy J. Mitchell

**Affiliations:** 1 Institute of Microbiology and Infection, College of Medical and Dental Sciences, University of Birmingham, Birmingham, United Kingdom; 2 Institute of Infection, Immunity and Inflammation, College of Medical, Veterinary and Life Sciences, University of Glasgow, Glasgow, United Kingdom; 3 Technology Hub Manager, Infrastructure and Facilities, College of Medical and Dental Sciences, University of Birmingham, Birmingham, United Kingdom; Oregon Health & Science University, UNITED STATES

## Abstract

Bioluminescence has been harnessed for use in bacterial reporter systems and for *in vivo* imaging of infection in animal models. Strain Xen35, a bioluminescent derivative of *Streptococcus pneumoniae* serotype 4 strain TIGR4 was previously constructed for use for *in vivo* imaging of infections in animal models. We have shown that strain Xen35 is less virulent than its parent TIGR4 and that this is associated with the expression of the genes for bioluminescence. The expression of the *luxA-E* genes in the pneumococcus reduces virulence and down regulates the expression of the pneumococcal pilus.

## Introduction

*S*. *pneumoniae* is normally found as part of the commensal flora in the nasopharynx of humans and is also the leading cause of bacterial pneumonia. Pneumococcal infection can also manifest as meningitis, septicaemia and otitis media. To study pneumococcal disease a well characterised murine model is widely used [1, 2]. Recent advances in the development of fluorescent and bioluminescent reporters have made it possible to visualise disease progression in animals in real time. This methodology also reduces animal numbers used.

Bioluminescence is generated by a chemical reaction which releases energy in the form of light [3]. This occurs naturally in several organisms. The bioluminescence reaction consists of oxidation of reduced flavin mononucleotide (FMNH2) and a long chain aldehyde (RCHO) to produce light, flavin mononucleotide (FMN), fatty acids (RCOOH) and water. Five genes are required for all bioluminescent reactions in all bacterial species [4]. These genes are *luxA*/*luxB*, which encode the luciferase enzyme and *luxC-E*, which encode the enzymes required for synthesis of the substrate for the luciferase [4]. Oxygen is also required for the enzymatic reaction to take place. Cloning of the *lux* genes into non-native organisms to make them bioluminescent has enabled this chemical reaction to be utilised as a tool to visualise bacteria.

*S*. *pneumoniae* is naturally competent making genetic manipulation of most strains relatively easy [5, 6]. To study pneumococcal disease progression *in vivo* using bioluminescence, a panel of bioluminescent pneumococcal strains, that are now commercially available, were constructed [7–9]. Initial construction was performed by placing *luxA-E* amplified from *Photorhabdus luminescens* (formerly known as *Xenorhabdus luminescens*) and a kanamycin resistance cassette onto a promoterless transposon, which was randomly inserted into the genome of serotype 2 strain D39 [7, 8]. Bacteria were screened for levels of bioluminescence and the most highly expressing strain was designated Xen7. The site of insertion of the *lux* genes was mapped and shown to be in the gene coding for a hypothetical protein of unknown function (Gene number SPD_1717). This mutation was then transferred into a panel of *S*. *pneumoniae* strains by transformation using genomic DNA isolated from Xen7 [8]. Transformation was initially performed into a serotype 4 strain TIGR4, which was the first strain of *S*. *pneumoniae* to undergo complete whole genome sequencing [10]. Transformation of TIGR4 with Xen7 DNA and selection of luminescent mutants gave rise to Xen35, which had *luxA-E* inserted into SP_1914 (the TIGR4 equivalent of SPD_1717). The stable integration of these genes into Xen35 removed the need for use of antibiotics *in vivo* to maintain a plasmid expressing these genes. The Xen strains and other bioluminescent strains have been used in a number of studies to evaluate virulence of *S*. *pneumoniae in vivo* [8, 9, 11–14].

A recent study showed that a constitutive expression of the genes required for bioluminescence in *Citrobacter rodentium* resulted in a competitive disadvantage in a mouse model of infection and impaired growth in minimal media [15]. We evaluated the phenotypic characteristics of Xen35 in a mouse model of infection and found that Xen35 was less virulent compared to the parent TIGR4. We therefore used genomic and transcriptomic analysis to assess any differences between the two strains that may explain the difference in virulence. We identified a large number of genomic changes that appeared to have come from the strain Xen7. There were also numerous changes in gene expression levels in Xen35 compared to TIGR4. We therefore constructed a TIGR4 strain that had only the desired *lux* insertion, allowing evaluation of the effect of expression of the *lux* genes alone on virulence. This showed that expression of the *lux* genes in TIGR4 resulted in changes in levels of expression of known virulence factors and changes in the virulence of TIGR4 similar to that observed with Xen35.

## Materials and methods

### Ethics statement

All *in vivo* experiments were carried out in accordance with the UK Animals Scientific Procedures Act 1986 and covered by an appropriate Home Office project licence. The study was approved by the ethics committee of Biomedical Support Unit (BMSU) at the University of Birmingham.

Mice were sacrificed at a well-defined clinical endpoint to reduce suffering. Sacrifice was performed by cervical dislocation while the animals were under terminal anaesthesia.

### Bacterial strains and culture conditions

*S*. *pneumoniae* strains used in this study are shown in S4 Table. Strains were grown as described in [16]. Briefly strains were grown on blood agar base (BAB) with 5% horse blood or in brain heart infusion (BHI) broth at 37°C with 5% CO_2_. Growth media was supplemented with kanamycin (400μg ml^-1^) for Xen35 and T4P strains. For experimental techniques all strains were grown to mid log (OD_600nm_ 0.6) unless otherwise stated.

### Pneumococcal growth curve

Pneumococcal growth curves were performed in BHI broth comparing the growth of TIGR4, TIGR4 P2 and Xen35. 1x10^6^ CFU of *S*. *pneumoniae* were resuspended in 200μl BHI in a 96 well plate. Absorbance (600nm) readings were taken every 20 minutes for 10 hours using a a FLUOstar OPTIMA (BMG) plate reader. Triplicate readings were taken and assays performed in triplicate. Graphical presentation was performed in Prism version 4.0b (GraphPad Software).

### *In vivo* analysis

All *in vivo* experiments were carried out in accordance with the UK Animals Scientific Procedures Act 1986 and covered by an appropriate Home Office project licence. Mice had food and water ad libitum and were kept at a constant room temperature of 20–22°C and with a 12h light/dark cycle. All mice used in this study were MF1 (Harlan, UK) 6–8 week old outbred female mice from (Harlan, UK). Mice were sacrificed at a well defined clinical endpoint to reduce suffering. Sacrifice was performed by cervical dislocation while under terminal anaesthesia.

#### Pneumonia infection model

Groups of twenty mice were inoculated intranasally with 5x10^6^ colony forming units (CFU) in 50μl, of either Xen35, TIGR4 or T4P2. Five mice from each group were sacrificed at 24hr, 48hr or 72hr post infection (PI) and five mice were monitored for survival. Mice were sacrificed at the designated time point and organs were removed (lungs, liver, spleen, brain), nasal wash and blood taken and viable counts performed using the Miles and Misra method [17]. Tail vein bleeds were also taken throughout and animal weights and clinical scores were recorded. Survival group mice were sacrificed once clinical symptoms had reached a designated clinical end point. Bacterial load in organs and blood were compared between mice infected with Xen35 and TIGR4 at varying time points. Graphical representation and statistical analysis was performed in Prism version 4.0b (GraphPad Software). Statistical analysis was performed using a non-parametric Mann-Whitney two sample rank test, significance P<0.05. This statistical test was also used for comparison of the percentage weight loss of the mice over time. Survival of mice infected with different strains was compared using a Kaplain Meier survival curve and analysed using a Log-rank Test (P<0.05). No statistical analysis was performed on data if group sizes were below 5.

Competition experiments were done by inoculating 5 MF1 mice as above intraperitoneally (IP) with 1x10^4^CFU of Xen35 and TIGR4 in 200μl given in a 1:1 ratio. Mice were sacrificed at 24hr PI and bacterial count enumerated from organs, blood and nasal wash. Mice were processed as above except that organs, blood and nasal wash were differentially plated onto BAB only and BAB with kanamycin to evaluate the ratio of TIGR4 to Xen35. Statistical analysis was performed as noted above.

### Whole genome sequencing

Genomic DNA was isolated from mid log phase cultures using a phenol chloroform extraction method. Pre-sequencing DNA was fragmented using a Bioruptor sonicator and processed using a TruSeq DNA library preparation kit (Illumina, USA). Quality and quantity was measured using the bioanalyser and Kapa library quantification kits (KAPA Biosystems, UK) respectively. Samples were loaded onto the flow cells at 12pM on the Illumina Cluster station and sequenced using the IlluminaGAII (Xen35) or IlluminaGAIIx (T4P2). Output files were processed with CASAVA and fastQ files were generated.

Data analysis was performed in CLC Genomics Workbench 4.5.1 (CLC bio). Xen35 genome sequence was reference assembled to TIGR4 genome sequence available at NCBI (NC_003028), followed by SNP and Indel analysis. A De Novo assembly was also performed assembling the reads against themselves to give an indication if regions of low coverage are too divergent from the reference to assemble or are deletions. If this was the case primers were designed to cover these regions and areas were PCR amplified and sequenced by traditional Sanger Sequencing. From this analysis a complete Xen35 genome was assembled. (Sequence data are available at NCBI Bioproject PRJNA421796)

### RNA sequencing

#### Total RNA isolation

Total RNA isolation was performed as described in [16]. Briefly pneumococcal strains were grown in triplicate. RNA extraction was performed using the RNeasy (QIAGEN) kit as per the manufacturer’s instructions. Following RNA extraction, a further DNase step was performed using TURBO-DNA free kit (Ambion) as per the manufacturer’s instructions.

#### mRNA purification

RNA-seq analysis was performed on TIGR4 and Xen35. A total of 20μg of total RNA was used per strain for Ribosomal RNA (rRNA) depletion. rRNA was depleted using the MICROB*Express*TM Kit (Ambion, Fisher Scientific, UK) as per manufacturer’s instructions. rRNA depletion samples were analysed on the Bioanalyser 2100 (Agilent, UK) and concentrations were measured using the nanodrop ND 1000 (Thermo Scientific, UK).

#### cDNA synthesis

DS-cDNA synthesis was performed using the superscript DS-cDNA synthesis kit (LifeTechnologies) as per the manufacturers instructions. Final cDNA was resuspended in 10μl DNase free water. cDNA concentration and quality was measure on the nanodrop ND 1000 and Bioanalyser 2100 respectively. 2μg of cDNA from each sample was sent for sequencing. cDNA was processed using a TruSeq DNA library preparation kit (Illumina, USA). Quality and quantity was checked using the bioanalyser and Kapa library quantification kits (KAPA Biosystems, UK) respectively. Samples were loaded onto the flow cells at 12pM on the Illumina Cluster station and sequenced using the IlluminaGAIIx. Output files were processed with CASAVA and fastQ files were generated.

RNA-seq analysis was performed in CLC genomics workbench 7.0.3. Sequence reads were aligned to both the TIGR4 genome sequence (NC_003028) and to Xen35 genome sequence (Accession number), to prevent any bias during alignment. Differential expression was assessed in the RNAseq CLC analysis pipeline using default settings. Reads were normalized based on RPKM values. Fold changes between the two samples were assessed and bonferroni multiple testing correction applied P<0.05. Gene lists showing a significant change over two fold were compiled from both analysis (S2 Table).

RNA sequencing data is available at NCBI Bioproject PRJNA421796.

### RT-PCR

cDNA synthesis and RT-PCR was performed as described in [16]. The primers used for RT-PCR analysis are listed in S5 Table. Real-time PCR was performed using FastStart Universal SYBR green master mix (ROX) (Roche) as per the manufacturers instructions. *gyrA* was used as an internal control to normalise for cDNA synthesis variations. Analysis was performed in Opticom Monitor^™^ version 3.1. Background was subtracted using No-Reverse Transcriptase controls and replicates grouped together with at least two replicates used for analysis. Data was analysed using the 2^-ΔΔC^_T_ method [18], graphical data representation was performed in Prism version 4.0b (GraphPad Software), each bar representing the sample replica means ± standard deviation.

### Western blotting

Western blot analysis of the pilus protein RrgB and GroEL was performed as described in [16]. For all western blots *S*. *pneumoniae* strains were grown to OD_600m_ 0.6 in BHI. The bacterial pellet was resuspended in PBS and sonicated. Protein concentrations were normalised and prepared in sample buffer. Primary antibody consisted of a 1/4000 dilution of either an in house Mouse Anti-RrgB or Rabbit Anti-GroEL (*E*.*coli*) pAb (Enzo Life Sciences, UK). Secondary antibody was a 1/20,000 dilution Goat Anti-Rabbit IgG HRP linked F(ab)_2_ (GE Healthcare) or Goat Anti-Mouse IgG HRP (Southern Biotech).

### Construction of TIGR4 promoter strains (T4P1-4)

PCR was performed using high fidelity DNA polymerase Phusion (NEB, UK), as per the manufacturers instructions. All primers used for the construction of the T4P1-4 are listed in S6 Table. Initial cloning steps consisted of cloning the *lux* genes (*luxA-E*) into plasmid pCEP2 [19] at restriction sites NcoI and BamHI. The *lux* genes were amplified from Xen35 [8] using primers PcepluxF and PcepluxR (S6 Table). Plasmid and *lux* genes were digested with NcoI and BamHI and subsequently ligated using T4 DNA ligase (NEB), resulting in the plasmid pCEP2lux. This construct contains the *lux* genes under the control of a maltose inducible promoter. However, when transferred into TIGR4 no bioluminescence was visualised, suggesting the maltose inducible promoter was not strong enough to drive expression of *luxA-E* enough for it to be visualised in our assays.

To remove the maltose inducible promoter a StuI restriction site was engineered upstream of the maltose inducible promoter allowing complete excision via restriction digest with NcoI and StuI. Site directed mutagenesis (SDM) was performed to create a StuI site in pCEP2lux using primers Puc18 LSD F and Puc18 LSD R (S6 Table). SDM was performed using using high fidelity DNA polymerase Phusion (NEB, UK) as per manufacturers’ instructions. The PCR product was subsequently digested with DpnI enzyme as per the manufacturers guide, removing all template DNA from the reaction. Transformation was performed into XL1-Blue competent *E*.*coli*, creating plasmid pC2LSD.

To select promoters to drive expression of the *lux* genes, RNA-seq data was used to identify genes highly expressed during *in vitro* growth in the pneumococcus. Genes with the highest expression (RPKM) values were identified and the upstream region assessed for the presence of a promoter sequence. Expression of surrounding genes was also taken into consideration as they may be expressed as part of an operon. Finally sequence regions upstream of chosen genes were further assessed for the presence of promoter sequences using BPROM-softberry program that detects potential sigma70 promoter recognition sites. All regions selected contained a putative sigma70 binding site. Information about chosen promoters is in S3 Table.

For promoter cloning pC2LSD was digested using restriction enzymes StuI and NcoI removing the maltose inducible promoter. Promoters were amplified from TIGR4 using primers in S6 Table, and digested with StuI and NcoI. Vector and insert were ligated as above and then transformed into XL1-Blue *E*.*coli*. Plasmids constructed were pC2LSD P1/P2/P3 and P4. Plasmids were then transformed into TIGR4 and integration into the genome at the position of SP_1886 was confirmed by PCR.

### Pneumococcal transformation

Pneumococcal transformation was performed as described in [16, 20], except CSP-2 was used. BAB plates supplemented with kanamycin (400μg ml^-1^). For transformation 1μg of DNA was used.

### Evaluation of bioluminescence

1x10^6^ CFU of *S*. *pneumoniae* were added in 20μl BHI to a black F96 MicroWellTM plate (Nunc, Fisher Scientific, UK) containing 180μl BHI, each strain was tested in triplicate. Luminescence was measured on a FLUOstar OPTIMA (BMG) plate reader taking readings every 20 minutes for 10 hours. Graphical presentation was performed in Prism version 4.0b (GraphPad Software), each data point representing the mean of the triplicate luminescence reading minus background.

### Flow cytometry

*S*.*pneumoniae* strains were grown to OD600nm 0.6 in 5ml BHI. Cultures were centrifuged at 4000g for 5 minutes and fixed in 2% paraformaldehyde for 1 hour at room temperature (RT). Cells were resuspended in FACS buffer 1 (1x PBS, 1% BSA) and blocked at 4°C overnight. For antibody staining 100μl of fixed bacterial suspension was centrifuged and resuspended in 500μl buffer 2 (1x PBS, 1% BSA, 0.05% Tween 20) and 1μl of each primary antibody was added (Anti-Mouse RrgB polyclonal (in house production) and Anti-Rabbit Type Serum 4 (Statens Serum Institute, Denmark)) and incubated at RT for 1 hour. Cells were washed once (buffer 2) and then resuspended in 500μl buffer 2. 1μl of each secondary antibody was added (Goat Anti-Rabbit IgG Allophycocyanin (APC) conjugate (Southern Biotech, USA) and Goat Anti-Mouse IgG Fluorescein (FITC) Conjugate (Southern Biotech, USA)) and incubated for 1 hour in the dark at RT. Cells were washed three times in 500μl buffer 2 and then resuspended in 500μl buffer 1.

Samples were run on a FACScalibur flow cytometer (BD biosciences, USA), samples were acquired in CellQuest-Pro software (BD biosciences, USA). FACS analysis was performed in FlowJo 9.4.10 for Macintosh (Tree Star, USA). Cells were gated initially on being capsule positive (APC+) and then gated on selecting RrgB positive (FITC+) and RrgB negative populations. Percentage RrgB positive and negative populations were compared between parent and mutant strains.

### Fluorescence microscopy

10μl of antibody stained bacteria preparation was pippetted onto a 76x22mm glass slide (Menzel Gl00E4ser, Germany) and a 22x22mm glass cover slip (Menzel Gläser, Germany) placed over the sample. Samples were heated for 60 minutes at 50°C, and observed at X40 and X100 magnification using a Zeiss Axioscope M1 fluorescence microscope with the correct filters. Images were analysed in Volocity software 6.0.1 (Perkin Elmer). Images were arranged in Adobe Photoshop elements 10.

### Hydrogen peroxide assay

Hydrogen peroxide assay was performed to assess the ability of the different strains to produce hydrogen peroxide. *S*.*pneumoniae* cultures were grown to OD600nm 0.6 in BHI, 180μl of culture were added in triplicate to a 96 well flat bottom plate. To this 20μl of 3mg/ml ABTS (2,2 azinobis (3-ethylbenzthiazolinesulfonic acid) diammonium salt, Sigma-Aldrich, UK), 0.2mg/ml HRP (Peroxidase from horseradish Type II, Sigma-Aldrich, UK) in a 0.1M sodium phosphate buffer pH7.0 was added. The assay was left to develop for 30 minutes and 24 hours at 37°C followed by centrifugation at 1500g for 3 minutes. 100μl from each well was removed and placed into a fresh 96 well plate. Absorbance was measured using a spectrophotometer at 540nm (FLUOstar OPTIMA, BMG). Hydrogen peroxide standards were used as a positive control by making serial dilutions of Hydrogen peroxide 30% w/v AnalaR NORMAPUR (VWR, UK) in BHI. Graphical presentation of hydrogen peroxide assay data was performed in Prism version 4.0b (GraphPad Software). Data presented was the absorbance at each time point from each strain tested. No statistical analysis was performed.

## Results

### *In vivo* comparison of TIGR4 and Xen35

*C*omparison of virulence of TIGR4 and Xen35 was initially performed in a mouse model of pneumonia. MF1 mice were inoculated intra-nasally with either TIGR4 or Xen35 and the time to a defined clinical endpoint was measured (survival group). In further groups mice were sacrificed at 24, 48, 72 hours post infection to identify changes in bacterial load in organs, blood and nasal wash at these time points between the two strains.

**Fig 1:**
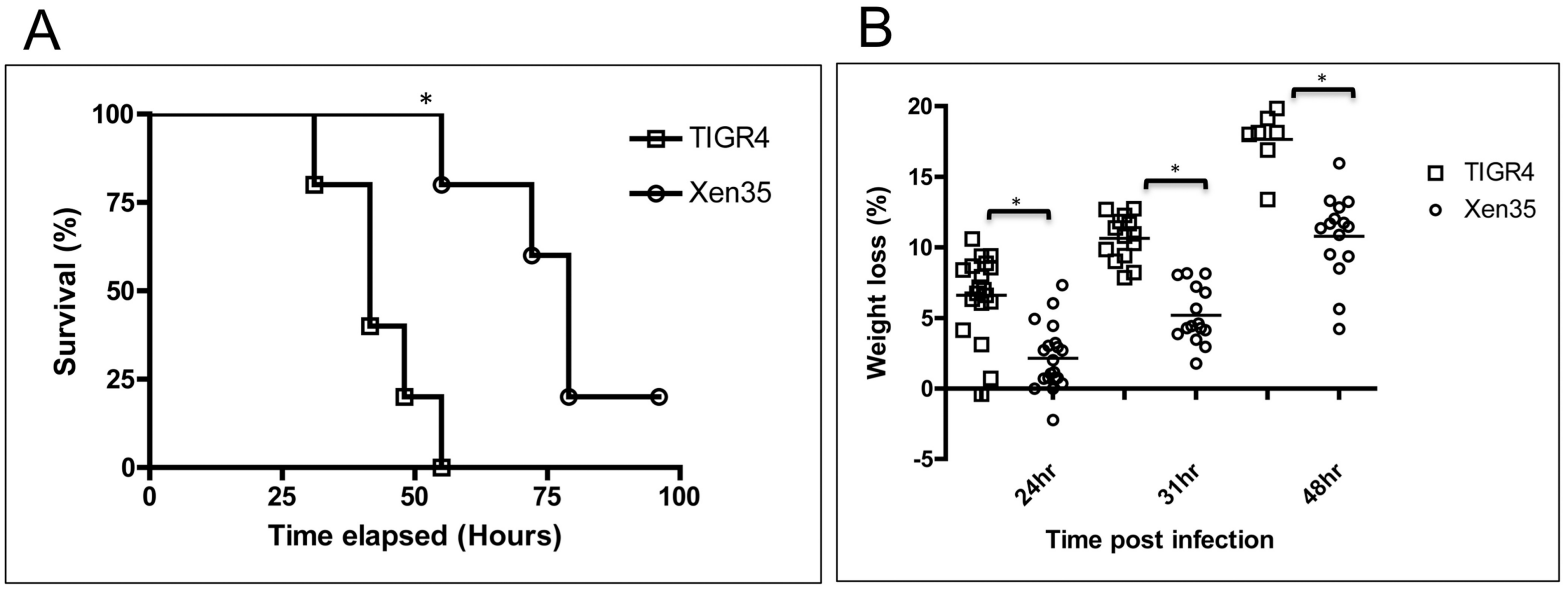
Survival and weight loss of mice infected with Xen35. (A) Percentage survival of mice intra-nasally inoculated with TIGR4 or Xen35 over time, statistical analysis was performed using a logrank Test, * P<0.01. * above the strain indicated a statistical difference compared to Xen35. Only the survival group was used for analysis (n = 5 per bacterial strain) (B) Percentage weight loss of mice infected with either Xen35 or TIGR4. All groups were used for analysis, later groups have smaller numbers due to some mice being sacrificed. Statistical analysis was performed using a non-parametric Mann-Whitney two sample rank test, *P<0.001.

A statistically significant increase in survival time of mice infected with Xen35 compared to TIGR4 was observed in the survival group (Fig 1A). Weight loss post infection was also used as a marker for disease progression and the percentage weight loss of mice infected with Xen35 was significantly lower at all time points than mice infected with TIGR4 (Fig 1B). Bacterial counts from the nasal wash in the 24 hour and survival group showed statistically lower counts in mice infected with Xen35 compared to TIGR4, however counts from other organs at all time were not significantly different (S1 Fig). No other significance was observed at later time points (S1 Fig). This may be due to the fact the TIGR4 infected group had to be sacrificed before 48 and 72 hours post infection. Therefore, only the 24 hour post infection group showed a true time comparison between the two strains.

**Fig 2:**
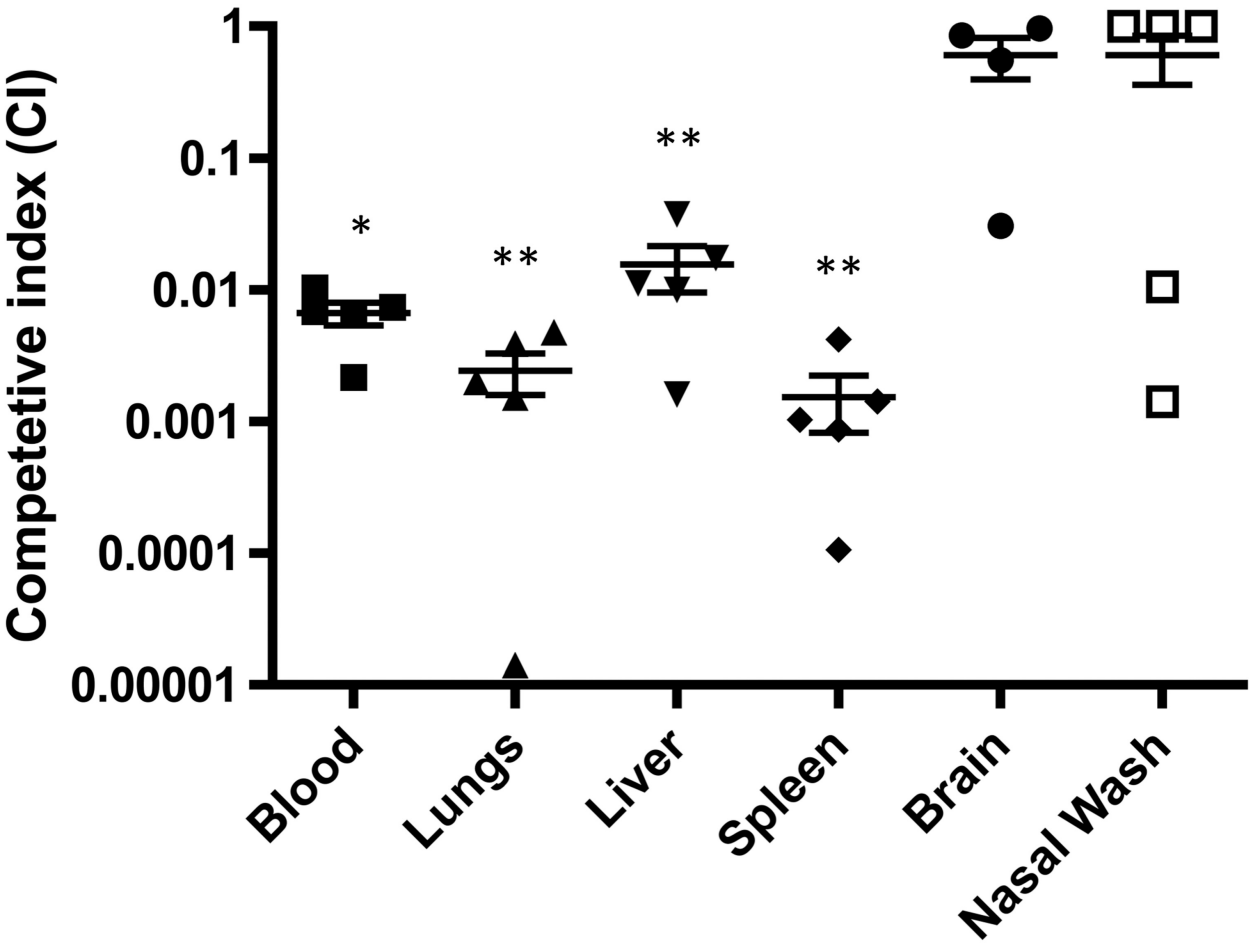
*In vivo* competition of Xen35 and TIGR4. Bacterial counts were enumerated from 5 mice at 24 hours post infection from mice infected via the IP route with a 1:1 mix of Xen35 and TIGR4. Bacterial counts were enumerated from brain, lungs, nasal wash, liver, spleen and blood for each strain by differential plating onto kanamycin. Statistical analysis was performed comparing TIGR4 and Xen35 raw counts using a non-parametric Mann-Whitney two sample rank test, *P< 0.05, ** P<0.01. Above is the competitive index calculated from the raw counts. */ ** above the organ/ body fluid shows a statistically lower bacterial count of Xen35 compared to TIGR4.

Competitive index was used to compare TIGR4 and Xen35 in a murine sepsis model. Mice were infected with a 1:1 ratio of TIGR4 and Xen35 given by the intraperitoneal route. Total bacterial organ loads were evaluated and Xen35 load was evaluated via selective plating on to kanamycin containing plates. Bacterial counts of Xen35 in the lungs, spleen, liver and blood were significantly lower than those of TIGR4 (Fig 2), indicating that Xen35 is less virulent than TIGR4 in a mouse model of disease.

### Whole genome re-sequencing of Xen35

To identify genomic changes that may explain the difference in virulence between the two “isogenic” strains whole genome sequencing was performed on Xen35. Xen35 sequence reads were assembled against the TIGR4 genome sequence (NC_003028). Comparison of Xen35 with TIGR4 revealed a total of 220 SNPs and 23 indels (Table 1 and S1 Table). The majority of these changes were surrounding the transposon insertion site (189 SNPs, 5 indels) corresponding to the recombination event that occurred when TIGR4 was transformed with Xen7 chromosomal DNA, during the construction of Xen35.

**Table 1:** Genome changes in Xen35 resulting in loss of function of the protein encoded or proteins containing large in frame deletions.

Gene	Function	Change	Outcome
SP_0200/0201	0200 Competence inducing protein Ccs4. 0201 Hypothetical protein	C deletion	0200- removal of stop codon. 0201- Frameshift and generation of premature stop codon
SP_0206	Hypothetical protein	C insertion	Frameshift. Removal of stop codon
SP_0491	Hypothetical protein	C deletion	Frameshift. Removal of stop codon
SP_0715	Lactate oxidase (LctO)	SNP (G>T	Glycine > stop codon
SP_0730	Pyruvate oxidase (SpxB)	SNP (G>T)	Glutamine > stop codon
SP_1715	Hypothetical protein	G Insertion	Frameshift. Premature stop codon
SP_1732	Serine/Threonine protein kinase (StkP)	216bp deletion	Romoval of PASTA domain 3 between amino acid repeats TQIVLTVAKKA/TVIVLTVAKKV
SP_1914	Hypothetical protein	*luxA-E* insertion	Gene loss of function
SP_1919	ABC transporter permease protein	SNP (TG>AA	Leucine to stop codon
SP_1920	MarR family transcriptional regulator	T+A deletion	Frameshift. Premature stop codon
SP2076	Authentic frameshift	G Insertion	Frameshift. Premature stop codon

Table compiling the changes in Xen35 that cause the gene encoded protein to become non-functional or to likely cause a reduced function.

**Fig 3:**
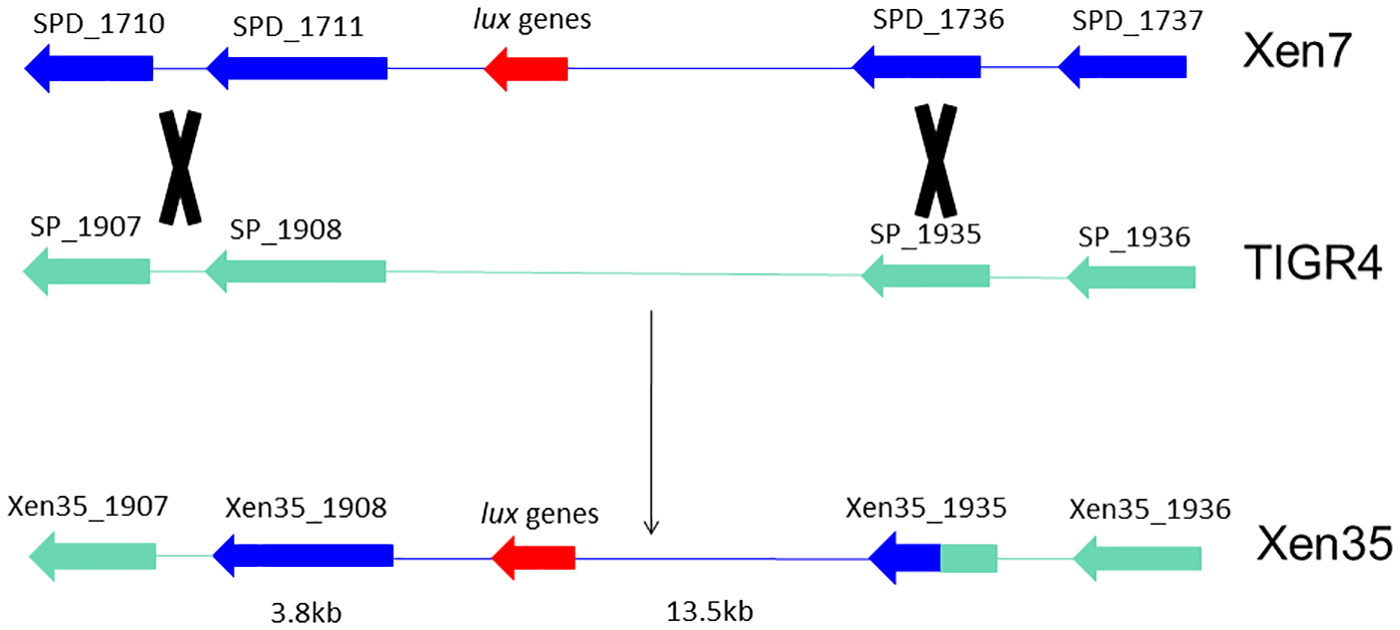
Schematic of recombination region in Xen35. Diagram shows region recombined into TIGR4 during transformation with Xen7 DNA. Dark blue indicates Xen7 DNA and green TIGR4 DNA. Along with the *lux* genes a large region spanning 17.3Kb was also recombined into TIGR4 creating Xen35.

By comparing the SNPs present in Xen35 to the TIGR4 and D39 (NC_008533) genome sequences, the site of the *lux* cassette recombination was mapped to between Xen35_1908 (3.8kb 5’ to the *lux* insertion) and Xen35_1935 (13.5kb 3’ to the *lux* insertion). The DNA in this region of the recombination in Xen35 is derived from Xen7 (Fig 3). The 5’ recombination event occurred between the end of Xen35_1908 and the intergenic region between SP_1907–1908, as the Xen35 sequence is identical to TIGR4 in the intergenic region (D39 shows 3 changes compared to TIGR4). The 3’ recombination event within Xen35_1935 contains a mixture of SNPs from TIGR4 and D39 therefore the recombination event took place within this gene. It is important to note that within this region there are some SNPs present in Xen35 that are not in either TIGR4 or D39. This is probably due to sequence variation of the D39 and TIGR4 strains used to make Xen7 and Xen35 compared to the sequences deposited in Genbank.

In the insertion region 100 SNPs are synonymous whereas 56 of the SNPs lead to 51 amino acid changes in 21 proteins, which may affect protein functionality. One of these SNPs introduces a premature stop codon in gene Xen35_1919 and one removes a stop codon in Xen35_1933. Only 2 of the indels in the *lux* recombination region are located in coding sequences. Both are located in Xen35_1920 (MarR transcriptional regulator) causing a frameshift mutation that disrupts the gene.

A further 31 SNPs and 18 indels were located outside this recombination region in Xen35 and were not introduced into TIGR4 with the *lux* genes. These changes are likely derived from the original TIGR4 strain used to construct Xen35. We do not have access to this parental strain for comparison. Within this study we compared Xen35 to TIGR4 obtained from a subculture of the original TIGR4 isolate that was genome sequenced (NC_003028).

For some of the major changes lying outside the *lux* recombination region further analysis was performed to assess if they were also recombined in from Xen7. For example, the deletion in *stkP* (SP_1732) was not present in Xen7 as determined by by PCR. Further the SNPs present in *spxB/ lctO* leading to truncated proteins and the lack of hydrogen peroxide production by Xen35 were not present in Xen7 and this strain produces normal levels of hydrogen peroxide. This suggests these changes are not the consequence of the transformation with chromosomal DNA from Xen7.

### Gene expression changes in Xen35

**Fig 4:**
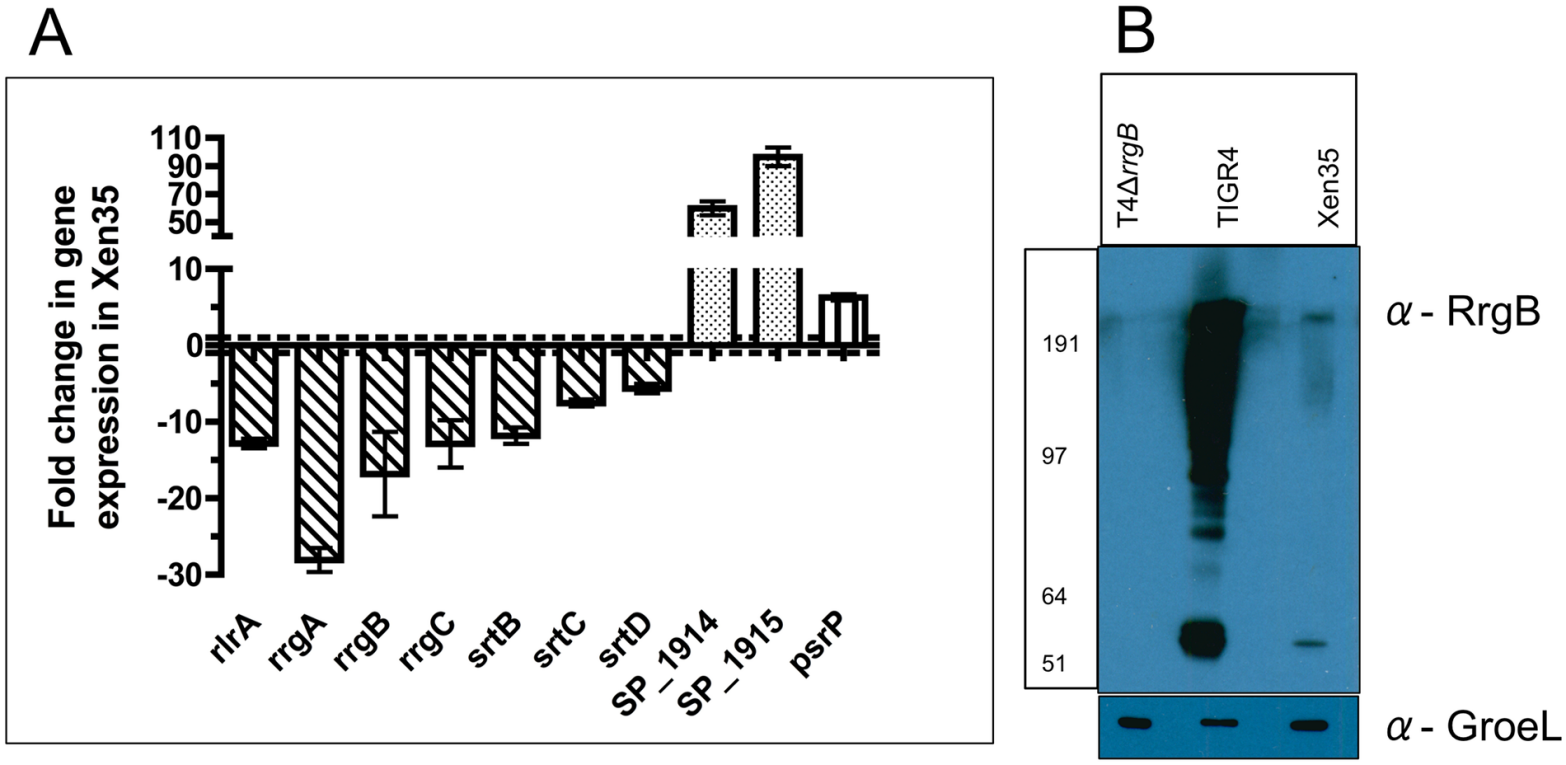
RT-PCR graph of pilus islet genes, *psrP* and SP_1914/15 expression in Xen35. (A) Graph shows RT-PCR of the whole pilus islet (*rlrA*, *rrgA*, *rrgB*, *rrgC*, *srtB*, *srtC*, *srtD*), *psrP*, SP_1914 and SP_1915 in Xen35. Fold change represents that of Xen35 compared to TIGR4. Each bar represents the average of at least two replicas and errors bars the standard deviation. (B) Western blotting analysis was performed on TIGR4, Xen35 and T4Δ*rrgB* looking at RrgB protein expression in all strains (α-RrgB antibody). Equal protein loading was confirmed by equal expression of GroEL (α-GroEL antibody).

Transcriptomic analysis was performed on Xen35 to evaluate any changes that may have been caused by the genomic changes. A total of 137 genes were differentially regulated in Xen35 compared to TIGR4 (S2 Table). Differentially regulated genes included those of known virulence factors including the down-regulation of the pilus genes, up-regulation of the serine rich repeat protein (*psrP*) and a two-component system regulator (RR09) [21–23]. Down-regulation of the genes for pyruvate oxidase (*spxB*) and lactate oxidase (*lctO*) was observed. These proteins are important during pyruvate metabolism and energy production. Further a number of genes involved in sugar utilisation and uptake were differentially regulated. These include the downregulation of the genes required for utlisation of fructose, malto-oligosaccharides and raffinose. In contrast in Xen35 there is an up-regulation of a PTS systems specifically for sucrose uptake and utilisation [24]. The genes surrounding the *lux* insertion site were also highly up-regulated in Xen35 compared to TIGR4, which explains the high level of bioluminescence in this strain. RT-PCR analysis was also performed to confirm some of the expression changes observed in the RNA-seq analysis (Fig 4A). Evaluation of the expression levels of the genes present on the pilus islet, *psrP* and the genes flanking the *lux* insertion confirmed the RNA-seq analysis. RT-PCR confirmed that all seven of the pilus islet genes were down-regulated in Xen35 and the genes surrounding the *lux* insertion (Xen35_1914/ Xen35_1915) were strongly up-regulated.

Western blot analysis was performed on Xen35 and TIGR4 to compare levels of the pilus backbone protein RrgB (Fig 4B). The decrease in transcription of the pilus islet shown via RNA-seq and RT-PCR also corresponds to a decrease in the amount of RrgB protein produced.

### Effects of expression of bioluminescence on TIGR4 pilus expression

The pneumococcal pilus is an important adhesin in the pneumococcus. The islet encoding the pilus has 7 genes, three coding for the structural pilus subunits (*rrgA*, *rrgB* and *rrgC*), one encoding a positive regulator of the operon (*rlrA*) and three encoding sortase enzymes involved in pilus assembly (*srtB*, *srtC*, *srtD)*. As described above, this islet is down regulated at the expression (RNA) and protein level (RrgB) in Xen35 (Fig 4A and 4B). In parallel with the effects of genome and expression changes seen in Xen35 we also evaluated the effect of expression of the *lux* genes on virulence and pilus expression in *S*. *pneumoniae*.

**Fig 5:**
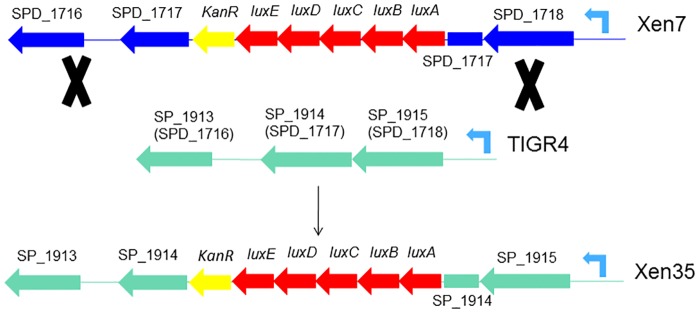
Schematic of l*ux* gene recombination site in Xen7 and Xen35. Diagram shows *lux* gene insertion site in Xen7 and recombination into TIGR4 creating Xen35. Dark blue indicates Xen7 DNA and green TIGR4 DNA. Red genes represent the 5 *lux* genes, yellow the kanamycin resistant cassette. In Xen7 these genes inserted in SPD_1717 making the gene non-functional. SDP-1717 homologue in TIGR4 is SP_1914 in which the *lux* genes are inserted in Xen35 making it non-functional. In light blue is the predicted promoter site located downstream of SPD_1717 and SP_1914 in Xen7 and Xen35 respectively. Thought to be pushing expression of the *lux* genes in both strains.

To test this a panel of bioluminescent *S*. *pneumoniae* strains were constructed that expressed the *lux* genes to different levels. Initial attempts were made to construct a bioluminescent TIGR4 strain by placing the *lux* genes in the same position as in Xen35. A PCR product containing the *lux* genes and flanking DNA was used to transform TIGR4 rather than the genomic DNA used in previous studies. The resultant strains transformed with a PCR product spanning genes Xen35_1917–1911 showed no bioluminescence despite successful recombination of the *lux* genes at the chosen region. This region contained a predicted promoter site downstream of SP_1915, which seems to be driving the expression of the *lux* genes in Xen35. Fig 5 shows gene orientation in this region and predicted promoter downstream of SP_1915. We assume this is the promoter driving expression of the *lux* genes in Xen35 as only SP_1914 and SP_1915 but not SP_1916 are up-regulated in the RNA-seq data (S2 Table).

An alternative method was therefore used to construct a panel of TIGR4 bioluminescent strains. Strong alternative promoters were chosen from analysis of the transcriptomic data to identify genes most highly expressed in TIGR4 under *in vitro* growth conditions (S3 Table). The promoters upstream of four highly expressed genes were cloned into a chromosomal expression platform (CEP) upstream of the *lux* genes in plasmid pCEP1. This plasmid contained the *lux* genes downstream of one of the four chosen promoters flanked by regions of pneumococcal genomic DNA that allow recombination into the expression locus in the TIGR4 genome [19]. The plasmids were designated pC2LSD P1, pC2LSD P2, pC2LSD P3 and pC2LSD P4 and on transformation into TIGR4, created strains T4P1, T4P2, T4P3 and T4P4. The CEP introduced the *lux* genes downstream of the promoters into a non-functional gene SP_1886 in TIGR4, and therefore should have no effects on the strain by insertion at this site [19]. The promoter upstream of SP_1915 was also used to evaluate the strength of the promoter driving expression of the *lux* genes in Xen35 (Fig 5). From this strain no bioluminescence was observed suggesting other genome changes are required for expression of the *lux* genes at the integration site in Xen35.

**Fig 6:**
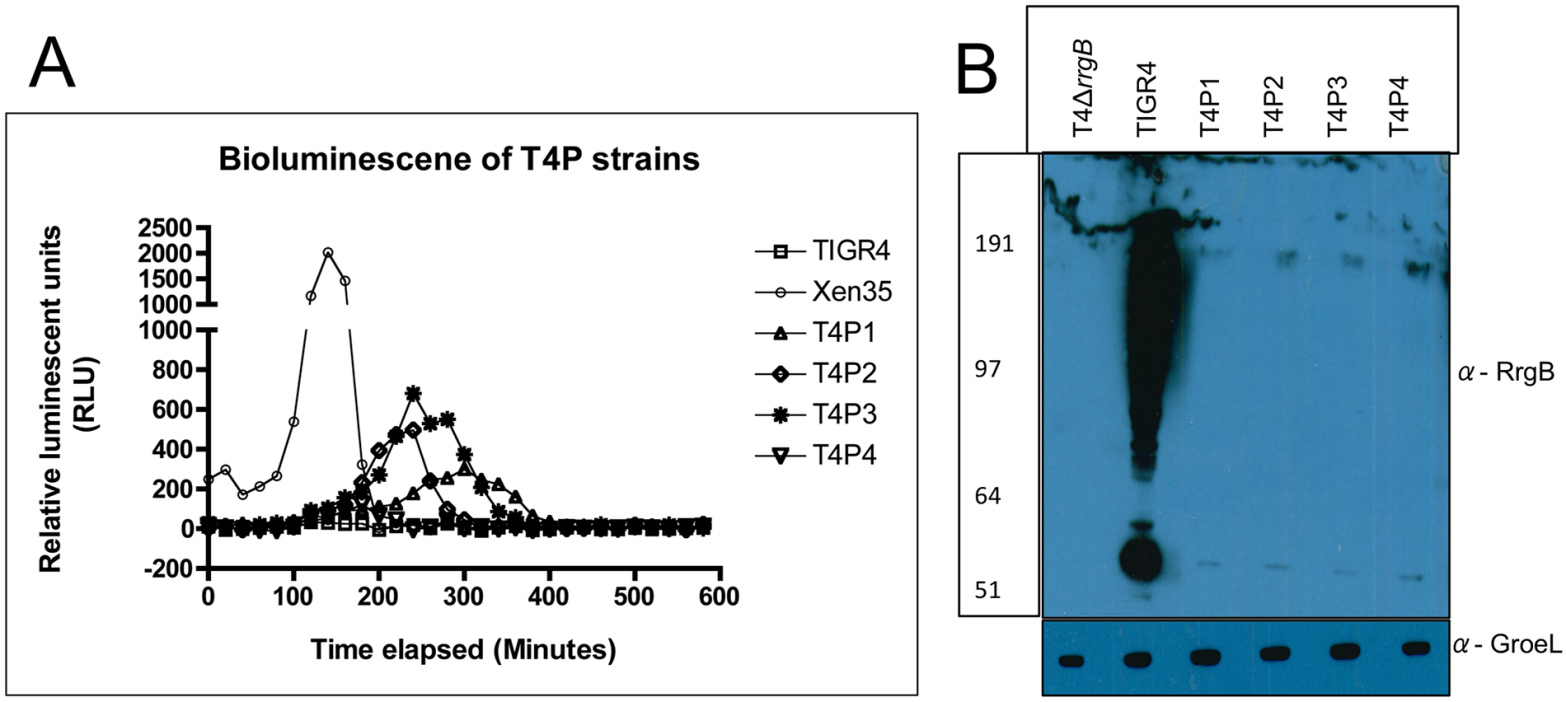
Bioluminescence of T4P strains. (A) Graph of bioluminescence of Xen35, TIGR4 and the T4P strains over time. Each point on the graph represents the average of a triplicate reading, Readings were taken every 20 minutes. (B) Western blotting analysis was performed on TIGR4, T4P1, P2, P3, P4 and T4Δ*rrgB* looking at RrgB protein expression in all strains (α-RrgB antibody). Equal protein loading was confirmed by equal expression of GroEL (α-GroEL antibody).

The level of bioluminescence of each strain was evaluated *in vitro* and compared to Xen35 (Fig 6A). Strains showed varying levels of bioluminescence compared to Xen35 due to the presence of the different promoters placed upstream.

Due to the observed differences in the pilus expression in Xen35 the level of the major pilus protein subunit (RrgB) was evaluated in the T4P strains (Fig 6B). Compared to TIGR4 all the TIGR4P strains showed severely reduced RrgB protein levels, similar to that observed in Xen35 (Fig 4). Virulence was also evaluated for the T4P strains using an intranasal infection model. All strains showed a trend to increased survival time compared to TIGR4 although not statistically significant due to the small number of animals used in this screen (n = 2) (S2 Fig).

**Fig 7:**
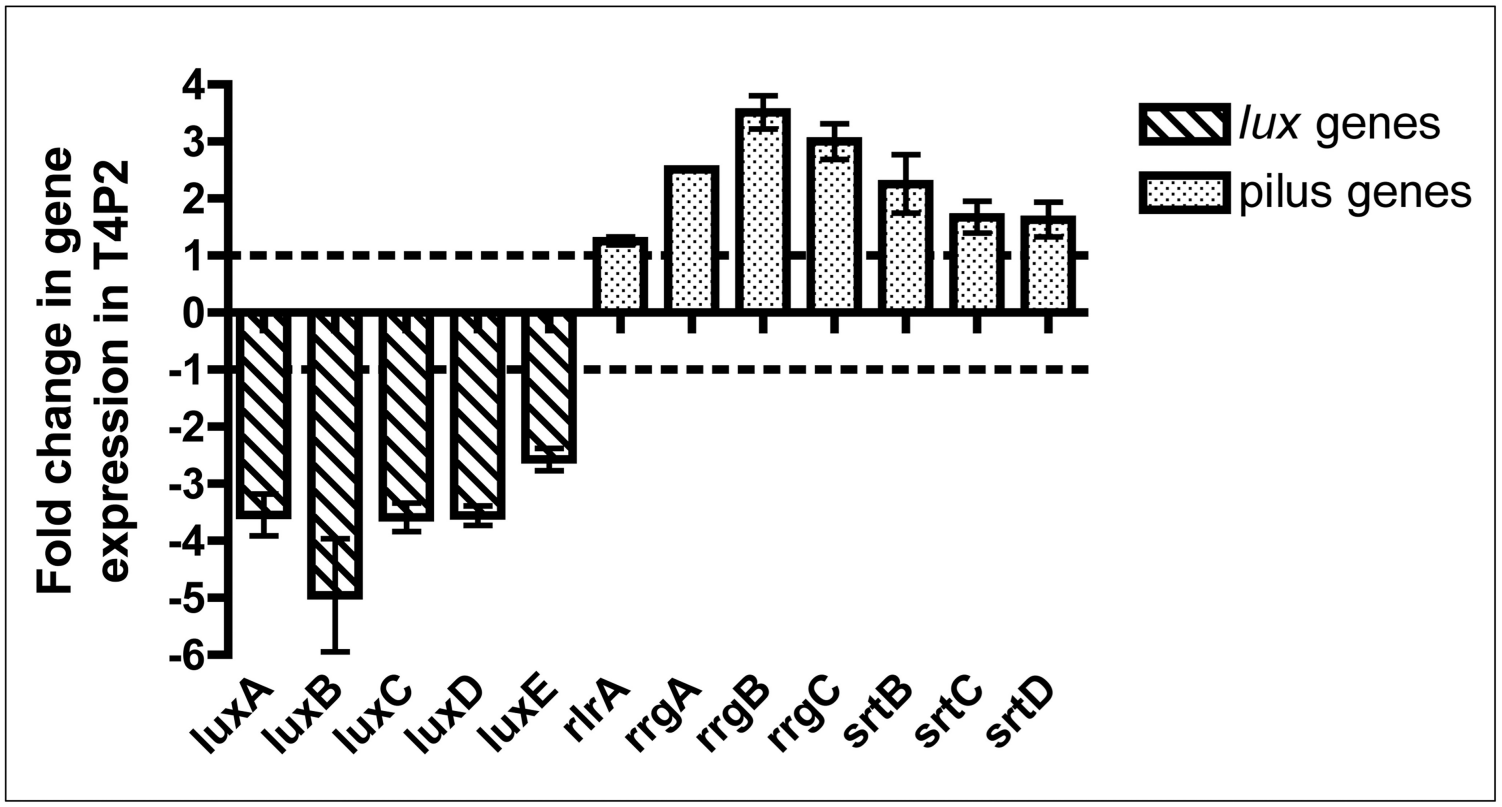
RT-PCR graph of *lux* and pilus genes expression in T4P2. Graph shows expression of *luxA-E* and the pilus islet genes (*rlrA*, *rrgA*, *rrgB*, *rrgC*, *srtB*, *srtC*, *srtD*) in T4P2. Fold change represents that of T4P2 compared to Xen35. Each bar represents the average of at least two replicas and error bars the standard deviation.

Strain T4P2 was chosen for further analysis and expression of the *lux* genes and pilus genes was evaluated by RT-PCR in relation to that observed in Xen35 (Fig 7). Compared to Xen35 T4P2 showed a 3–4 fold down regulation of the *lux* genes, which matches the levels of bioluminescence for T4P2 relative to Xen35 (Fig 6A). There was also a 2–4 fold up-regulation of the pilus islet genes in T4P2 compared to Xen35. This suggests the level of bioluminescence inversely correlates to the expression level of the pilus genes. High level expression of the *lux* genes correlated to low pilus expression levels. This suggests the bioluminescence genes may pose a metabolic burden on the bacterial cell. Pilus expression was also analysed at the population level in these two strains by measuring the cell surface expression of RrgB by flow cytometry (S3 Fig). This showed only 5.35% of Xen35 bacterial cells express RrgB on the cell surface. This is higher in T4P2 with 7.6% of bacteria expressing RrgB on the cell surface. Both are lower than TIGR4, which shown 87.9% of cells express RrgB on the cell surface. Fluorescence microscopy was used to validate antibody straining (S3 Fig).

**Fig 8:**
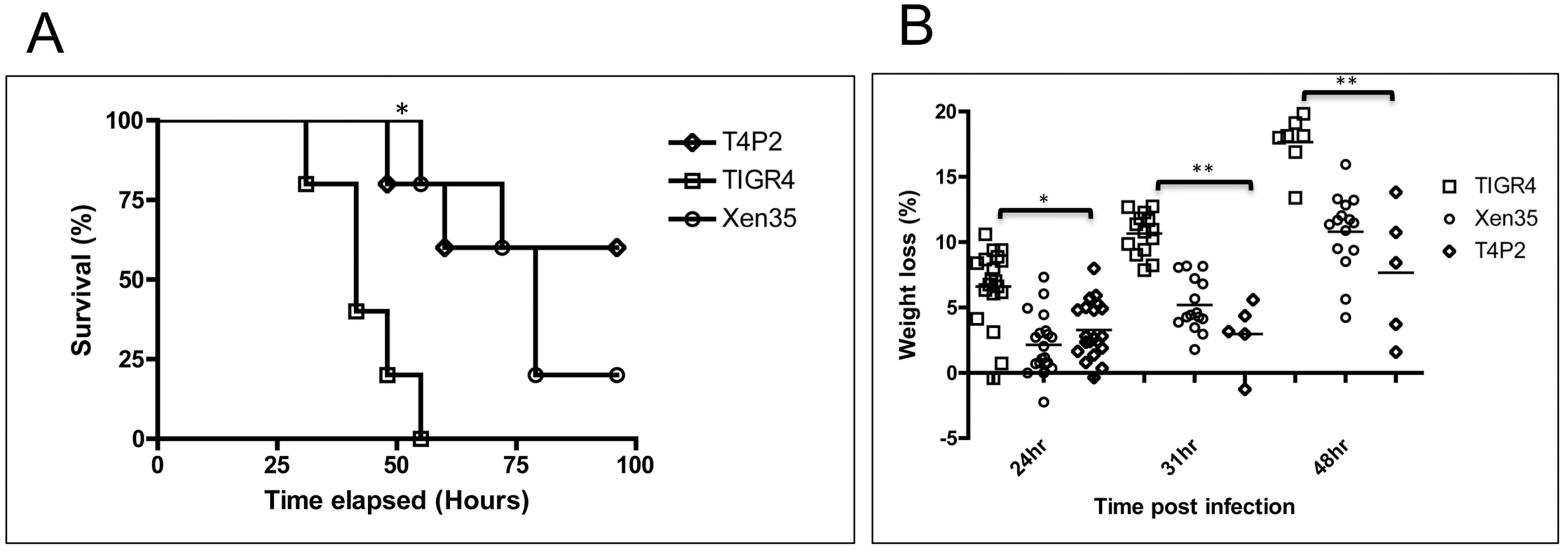
Survival and weight loss of mice infected with T4P2. (A) Shows percentage survival of mice infected with TIGR4, Xen35 or T4P2 over time, statistical analysis was performed comparing T4P2 to TIGR4 or T4P2 to Xen35 using a logrank Test, * P<0.01. * above the strain indicated a statistical difference compared to T4P2. Only the survival group was used for analysis (n = 5 per bacterial strain) (B) Shows percentage weight loss of mice infected with Xen35, TIGR4 or T4P2. All groups were used for analysis; later groups have smaller numbers due to some mice being sacrificed. Statistical analysis was performed using a non-parametric Mann-Whitney two sample rank test, *P< 0.01 and **P<0.001.

A larger study was done evaluating in a mouse model of infection the effect of expression of the *lux* genes on virulence in T4P2. Survival was monitored and percentage weight loss used as a gauge for disease progression (Fig 8). Survival of mice infected with T4P2 was statistically longer than those infected with TIGR4. Percentage weight loss was also significantly lower in mice infected with T4P2 compared to TIGR4. This effect is similar to that observed when using Xen35 (Fig 1).

To confirm that it is the expression of the *lux* genes and not genomic changes causing the noted effects, whole genome sequencing was performed on T4P2. The genomic changes observed are shown in Table 2. Only three genomic changes were observed in T4P2 compared to the TIGR4 genome sequence available. One was the insertion site where the *lux* genes were placed in T4P2. The other two remaining SNPs were both present in intergenic regions. Both changes observed have been seen in other serotype 4 TIGR4 strains and are likely a common genome change observed when compared to the genome sequence deposited in Genebank in 2008.

**Table 2:** Preliminary whole genome sequence changes in T4P2.

Gene	Information	Position TIGR4	Variant	
INT SP_1199–1200			Low coverage	
INT SP_1777–1778		1696086	Indel	T
SP_1886	New *lux* gene insertion site		Low coverage	

## Discussion

The bioluminescent strain Xen35 has been used to assess the virulence of TIGR4 *in vivo* and evaluate genes in TIGR4 important to virulence [8, 9]. In the current studies we observed clear differences in the virulence of Xen35 compared to TIGR4. This may be in part due to the large genomic changes observed in this strain compared to TIGR4 or due to the expression of the bioluminescence genes. *S*. *pneumoniae* has high rates of recombination and its genome can vary by 20–33% between different pneumococcal strains and sequence types [25]. There is also variation of strains of the same ST and serotype and these have been shown to varying greatly in their ability to cause disease [26, 27].

In strain Xen35 (parent TIGR4) the *lux* genes were inserted into SP_1914 along with large flanking regions isolated from strain Xen7 (parent D39) [7, 8]. These genes in Xen35 are therefore of a D39 (serotype 2) allele rather than a TIGR4 (serotype 4) allele. Although the majority of these changes introduced are synonymous, a number of them result in predicted amino acid changes in the expressed proteins. Of the genes recombined in with the *lux* insertion some have been implicated to play a role in virulence including, SP_1909, SP_1913, SP_1923 and SP_1931 and may contribute to the reduced virulence observed in Xen35 [28]. Whether these changes affect the functionality of the protein is unknown. However, genes in different strains have been shown to act in a strain specific manner and therefore these changes may contribute to changes in virulence of Xen35 compared to TIGR4 [20]. A number of genomic changes in Xen35 were also located outside of the *lux* gene recombination region. Some of these changes could also be mapped to genomic changes in D39 (NC_008533), indicating perhaps multiple recombination events during a single transformation, which has been noted previously [29]. Some of these changes are located in genes known to be important for virulence and include SP_0784, SP_0807, SP_0927, SP_1343 and SP_1891 [11, 28, 30]. A number of changes also result in disruption of genes known to code for virulence factors including SP_0200, SP_0201, SP_0206, SP_0730, SP_1715 and SP_2076 [28].

Other changes in Xen35 include an in-frame deletion of the N-terminal region of the serine threonine protein kinase (StkP) that removes the third penicillin binding protein and serine/threonine kinase associated (PASTA) domain. StkP has been shown to be important for virulence in models of bacteraemia and pneumonia [16, 28, 31, 32]. These domains have also been shown to be important for the normal functioning of StkP and deletion of one domain alters StkP signalling capabilities [16, 33]. This deletion has been shown to modulate expression of the pilus in TIGR4 [16] but does not explain the altered pilus expression in the Xen35 background. StkP acts to repress the pilus [16] therefore deletion of the PASTA domain is predicted to cause an increase in pilus expression rather than the decreased expression observed in Xen35, suggesting that other changes in Xen35 override the role of StkP in regulation of the pilus.

Xen35 also contains SNPs that introduce premature stop codons in the *spxB* and *lctO* genes, which results in Xen35 producing no hydrogen peroxide (S4 Fig). The *spxB* gene has been shown to play a role in colonisation and to a lesser extent a role in replication in the lungs and translocation to the bloodstream [9, 34]. This gene is also important in the production of hydrogen peroxide as a by-product of its enzymatic reaction, which allow the pneumococcus to compete with other colonising bacteria in the nasopharynx [35]. Although these genes are important for virulence in the pneumococcus we don’t believe these are the cause for the reduced virulence observed in Xen35 and T4P2, as Xen35 produces no hydrogen peroxide yet, T4P2 produced similar levels of hydrogen peroxide to TIGR4 (S4 Fig).

The sequencing data shows the occurrence of several important mutations that are predicted to contribute to the loss of virulence of Xen35. Some of these mutations may have been selected for during the screening process on the basis that they increase light production. In addition to the mutations in the genome and associated expression changes observed in Xen35, it is clear that expression of the *lux* genes themselves alters virulence (Fig 8). Strain T4P2 that is TIGR4 expressing *lux* from promoter P2 but is otherwise isogenic has reduced virulence in the mouse model when compared to TIGR4. There is a correlation between expression of the *lux* genes and expression of the pilus islet genes. Increased expression of the *lux* genes is associated with a decrease in expression of the pilus islet genes (Fig 7). Pilus regulation has been shown to occur at the population level [36, 37]. The reduction in pilus expression in Xen35 and T4P2 was also observed at the population level with Xen35 only showing 5% of bacterial cells expressing the pilus on the cells surface. This was slightly higher in T4P2, confirming our findings that there is increased pilus expression in T4P2. It is possible that changes in expression of other genes shown to be differentially regulated in Xen35 (S2 Table) are also due to expression of the *lux* genes alone.

We observed no difference in growth rate of TIGR4 and T4P2 (S5 Fig), however Xen35 showed a clear reduction in growth rate when grown in complete medium (BHI). Xen35 produces significantly more bioluminescence than T4P2 (Fig 6A) and it may be in T4P2 this lower level of bioluminescence is not enough to cause the growth defects observed in Xen35. The fact we see pilus expression levels are dependent on the level of bioluminescence shows the effect of bioluminescence expression has a dose dependent effect on the bacterial cell (Fig 7). If nutrient depleted media were used potential differences in these strains may become more evident. This would mimic more conditions in the animal model where nutrients are less abundant. In this model we saw a clear difference in virulence of T4P2 and Xen35 strains compared to TIGR4, suggesting in this environment the expression of the *lux* genes poses a metabolic burden on the cell.

**Fig 9:**
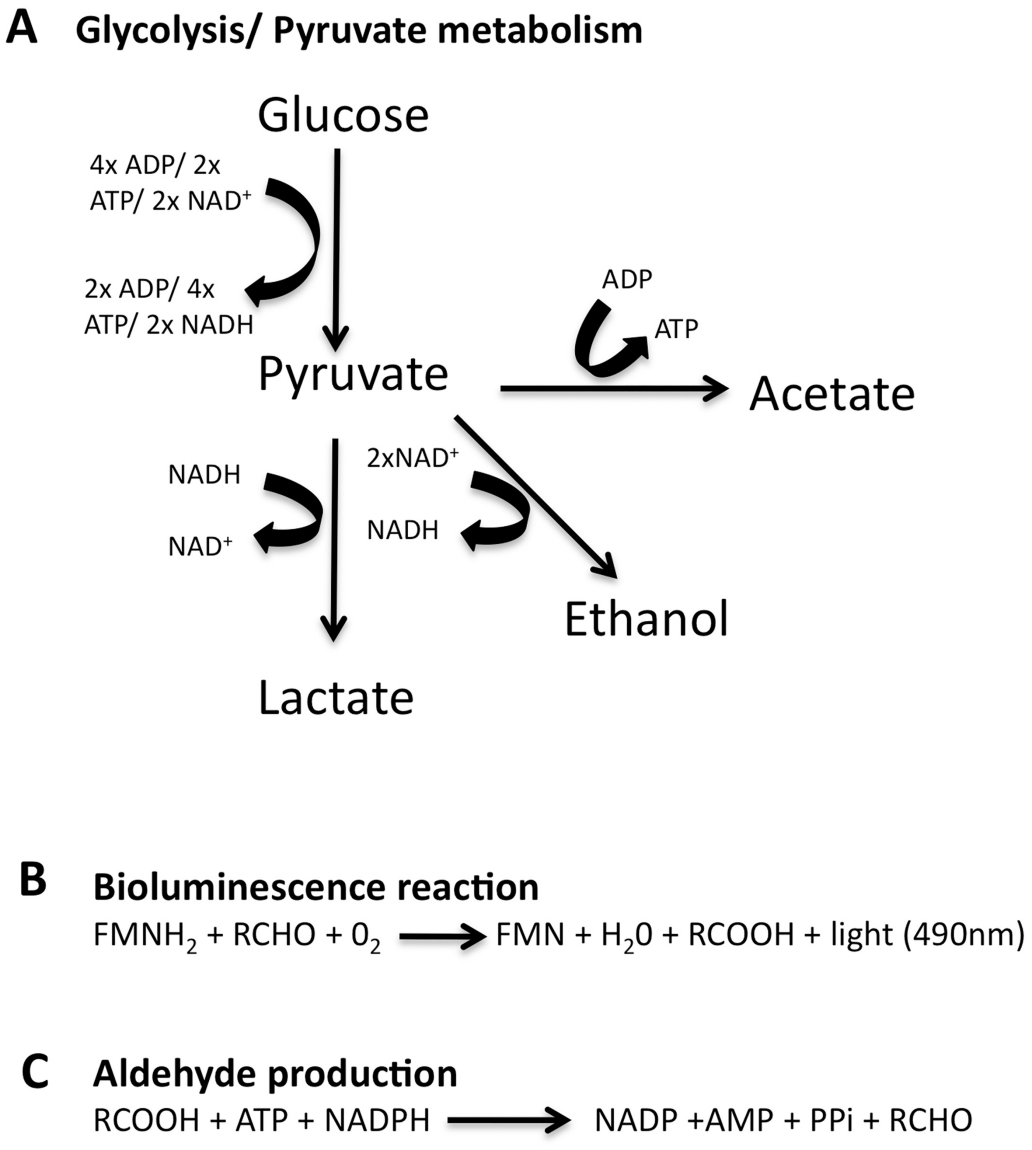
Energy production and consumption. (A) Reaction catalysed by luciferase enzyme (Flavin reductase) leading to light emission (bioluminescence). Reaction requires reduced riboflavin (FMNH_2_), long chain aldehyde (RCHO) and oxygen (O_2_) resulting in flavin mononucleotide (FMN), Water (H_2_O), fatty acids (RCOOH) and light [3] (B) Diagram showing adenosine triphosphate (ATP), reduced nicotinamide adenine dinucleotide (NADH), adenosine diphosphate (ADP) and oxidised nicotinamide adenine dinucleotide (NAD^+^) production and utilisation during glycolysis and pyruvate metabolism in *S*. *pneumoniae* [46]. (C) Reaction catalysed by the fatty acid reductase complex required to produce the long chain aldehyde substrate for the luciferase enzyme. Reaction requires RCOOH, ATP, reduced nicotinamide adenine dinucleotide phosphate (NADPH) resulting in nicotinamide adenine dinucleotide phosphate (NADP), adenosine monophosphate (AMP), pyrophosphate (PPi) and RCHO [3].

Naturally bioluminescent bacteria are metabolically equipped to produce the substrates required for the bioluminescence reaction and the energy requirements this needs. However, in normally non-bioluminescent bacterial species expression of the *lux* genes may lead to alterations in normal cell function and virulence. This has been shown before in the gram- negative pathogen *C*.*rodentium*, where expression of the *lux* genes resulted in reduced virulence in a mouse model of infection [15]. Potential causes of this may be altered redox balance within the cell as the chemistry of light production requires large amounts of reducing power for the recycling of FMN to FMNH_2_ (Fig 9A) [38, 39]. FMNH_2_, is oxidised during light production giving FMN. Recycling between FMN and FMNH_2_ is catalysed by flavin reductase and is NAD(P)H dependent [38, 40]. The *lux* gene cassette introduced to construct bioluminescent strains does not contain flavin reductase and this function must be performed by a host enzyme. In the pneumococcus the NADH dependant flavin reductase converts FMN to FMNH_2_ and results in oxidation of NADH to NAD^+^. Potentially altering the levels of NAD^+^ relative to NADH within the bacterial cell. The ability to maintain the redox balance within the cell is vital for proper cellular growth and functioning. NADH and NAD^+^ are required during carbon catabolism and the constant recycling between the two factors must be maintained. During glycolysis NAD^+^ is oxidised producing its reduced equivalent NADH, which is recycled back to NAD^+^ during fermentation of pyruvate to lactate (Fig 9B). Changes in redox balance have been shown to cause variation in Gram-positive and Gram-negative bacterial metabolism [41–45]. Disruption in catabolism has been shown in the pneumococcus to alter regulation of large groups of genes, altering virulence and regulation of virulence factors [46, 47]. The gene expression reported here support that this may be the case in Xen35 as shown by the down regulation of a number of sugar transporters and the up-regulation of a sucrose specific PTS system in Xen35. Variations in redox balance may also affect the functionality of NADH dependant enzymes such as NADH oxidase in the pneumococcus, which is important in helping prevent the toxic effects of oxidative stress on the cell [48].

Levels of ATP may also be compromised through the utilisation of ATP for production of the aldehyde substrate required for bioluminescent reaction (Fig 9C). The substrate for the luciferase enzyme is a long chain fatty aldehyde, which is produced by the fatty acid reductase complex encoded by *lux*CDE. The majority of the ATP produced within the pneumococcus is synthesised under aerobic conditions via pyruvate oxidase (SpxB) and acetate kinase (AckA), resulting in acetate production [49, 50]. Depletion of ATP may also have an effect on cellular metabolism as it is required during glycolysis. There are no genes present in the pneumococcus encoding the enzymes for the TCA cycle or electron transport chain [10] and the production of energy in the pneumococcus is strictly fermentative via glycolysis followed by pyruvate metabolism to lactate, acetate, formate or ethanol [51]. Glycolysis yields two ATP molecules, two NADH molecules and two molecules of pyruvate. Pyruvate fermentation follows glycolysis, under both anaerobic and aerobic conditions. During fermentation pyruvate is converted to lactate via lactate dehydrogenase (NADH dependant), simultaneously NADH is converted to NAD^+^, which is required for glycolysis. Evidence for depleted ATP stores within the bioluminescent strain Xen35 is the presence of a SNP in *spxB*, resulting in a non-functional gene. Mutations in acetate kinase (AckA) have been shown to be unstable and result in spontaneous mutation in *spxB* or *spxR* (SpxB regulator) [49]. This is proposed to be due to the requirement for ATP to repair the cellular damage created by the production of hydrogen peroxide. This may explain the presence of SNPs in the *spxB* gene of Xen35 as it is unable to make sufficient ATP to repair the cellular damage induced by hydrogen peroxide.

The reduced virulence observed in both Xen35 and T4P2 is likely a result from both of the points discussed above and this has a global effect on the bacteria, placing a large stress on the cell. The effect of expression of bioluminescence genes on virulence may also be applicable to other bacterial species that are not genetically equipped to deal with requirements of the chemistry of light production.

This study provides an insight into the potential for genomic changes to be incorporated into strains during transformation. Further we have shown that the cloning of non-native genes may alter virulence of the parent strain making it difficult to use these manipulated strains in virulence studies. For the future, strains that have been genetically manipulated in this manner should be evaluated as to what effect is has on the strain prior to use in virulence studies.

## Supporting information

S1 Fig Bacterial counts in organs and bodily fluids of MF1 mice infected with Xen35 or TIGR4. Bacterial counts were enumerated from brain, lungs, nasal wash and blood of mice infected intranasally with either Xen35 or TIGR4. Counts were enumerated from 5 mice for each strain over varying time points, graph (A) represent the 24 hour post infection time point, (B) 48 hours, (C) 72 hours and (D) the survival time point. Statistical analysis was performed using a non-parametric Mann-Whitney two sample rank test, *P< 0.05.
(TIF)

S2 Fig Survival of mice infected with T4P strains. Shows percentage survival of mice infected intranasally with T4P strains over time compared to Xen35 and TIGR4 (n = 2/ bacterial strain). No statistical analysis was performed due to the small number of mice used per group.
(TIF)

S3 Fig RrgB surface expression in Xen35 and T4P2. FACS was performed on TIGR4, T4P2, Xen35 and T4Δ*rrgB*. Samples were initially gated on for being capsule positive using a type 4 antisera followed by a secondary APC conjugate. These were then gate on for being RrgB positive (A) Shows histograms/ polychromatic plot of bacteria stained with an RrgB primary antibody followed by FITC secondary. RrgB negative population is shown on the left and positive on the right. Table shows the percentage RrgB positive and negative cells for each strain when a gate was set on the TIGR4Δ*rrgB* control to include less than 2% of events. (B) Fluorescence microscopy was performed on all FACS samples to confirm correct antibody staining. All fluorescence microscopy images were taken at X40 and X100 magnification using a Zeiss AxioscopeM1 fluorescence microscope. One representative image is shown for each bacterial strain. Further images can be seen in S6 Fig.
(TIF)

S4 Fig Hydrogen peroxide production of Xen35 andT4P strains. Each strain was represented in triplicate in the Hydrogen peroxide assay. (A) The graph gives the absorbance at 540nm at 30 minutes and 24 hours post incubation with the chromogenic substrate (B) Shows visually the assay performed in a 96 well plate at 24 hours post incubation.
(TIF)

S5 Fig Growth of TIGR4, Xen35 and TIGR4 in complete media. Graph of growth rates of TIGR4, Xen35 and T4P strains over time when grown in BHI broth. Each point on the graph represents the average of a triplicate experiments. Absorbance readings (600nm) were taken every 20 minutes for 10 hours.
(TIF)

S6 Fig Florescence microscopy images of bacterial RrgB cell surface expression. Three to four representative image of fluorescently labelled TIGR4 (A), T4Δ*rrgB* (B), Xen35 (C) and T4P2 (D) used for FACS analysis. Cells were stained for the presence of RrgB (FITC) and the capsule (APC). All fluorescence microscopy images were taken at X40 and X100 magnification using a Zeiss AxioscopeM1 fluorescence microscope.
(TIF)

S1 Table Whole genome change in Xen35 compared to TIGR4. Table lists all 243 genome changes in Xen35 compared to TIGR4. Information includes the type of change with three types of SNP, SNP-NS (NON SYNONYMOUSE SNP), SNP-S (SYNONYMOUSE SNP) and SNP-D (Dynamic SNP, mix of both). Position of change is included in the TIGR4 genome sequence data and the Xen35 genome sequence data. Changes are also stated whether it correspond to that of D39 or neither (not in D39 or TIGR4).
(DOCX)

S2 Table Transcriptomic analysis of Xen35 compared to TIGR4. RNA-seq gene expression changes observed in Xen35 compared to TIGR4. Table shows list of significantly differentially regulated genes with over 2 fold change. All genes were found to be differentially regulated when data was aligned to TIGR4 and Xen35 independently, With the exception of SP_1915 and SP_0517, which were only differentially regulated when data was aligned to TIGR4. Fold change represent the average fold change between the two analyses bar the two genes noted above.
(DOCX)

S3 Table Table of promoters used to drive expression of the *lux* genes. (DOCX)

S4 Table Names of all *S*. *pneumoniae* strains used in this study. Table of all *S*. *pneumoniae* strains used in this study with their antibiotic profiles. All strains unless a reference is given were constructed by the author.
(DOCX)

S5 Table Primers and related information used for RT-PCR analysis. (DOCX)

S6 Table Table of primers used for construction of T4P strains. (DOCX)
