# Supplementary figures and images for: Expression of the lux genes in Streptococcus pneumoniae modulates pilus expression and virulence
Source: PLoS One. 2018 Jan 17;13(1):e0189426. doi: 10.1371/journal.pone.0189426 (PMC5771582; doi:10.1371/journal.pone.0189426)

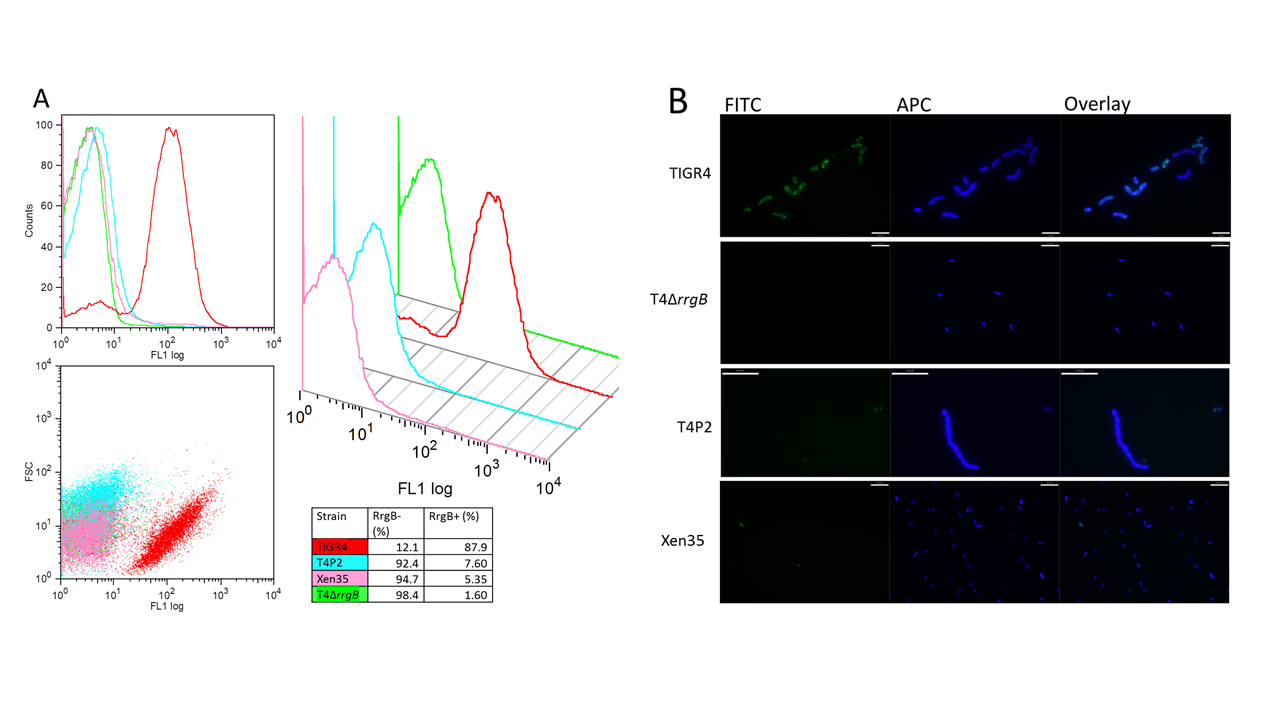

Supplement: S3 Fig — FACS was performed on TIGR4, T4P2, Xen35 and T4ΔrrgB. Samples were initially gated on for being capsule positive using a type 4 antisera followed by a secondary APC conjugate. These were then gate on for being RrgB positive (A) Shows histograms/ polychromatic plot of bacteria stained with an RrgB primary antibody followed by FITC secondary. RrgB negative population is shown on the left and positive on the right. Table shows the percentage RrgB positive and negative cells for each strain when a gate was set on the TIGR4ΔrrgB control to include less than 2% of events. (B) Fluorescence microscopy was performed on all FACS samples to confirm correct antibody staining. All fluorescence microscopy images were taken at X40 and X100 magnification using a Zeiss AxioscopeM1 fluorescence microscope. One representative image is shown for each bacterial strain. Further images can be seen in S6 Fig. (TIF) [file pone.0189426.s003.tif]

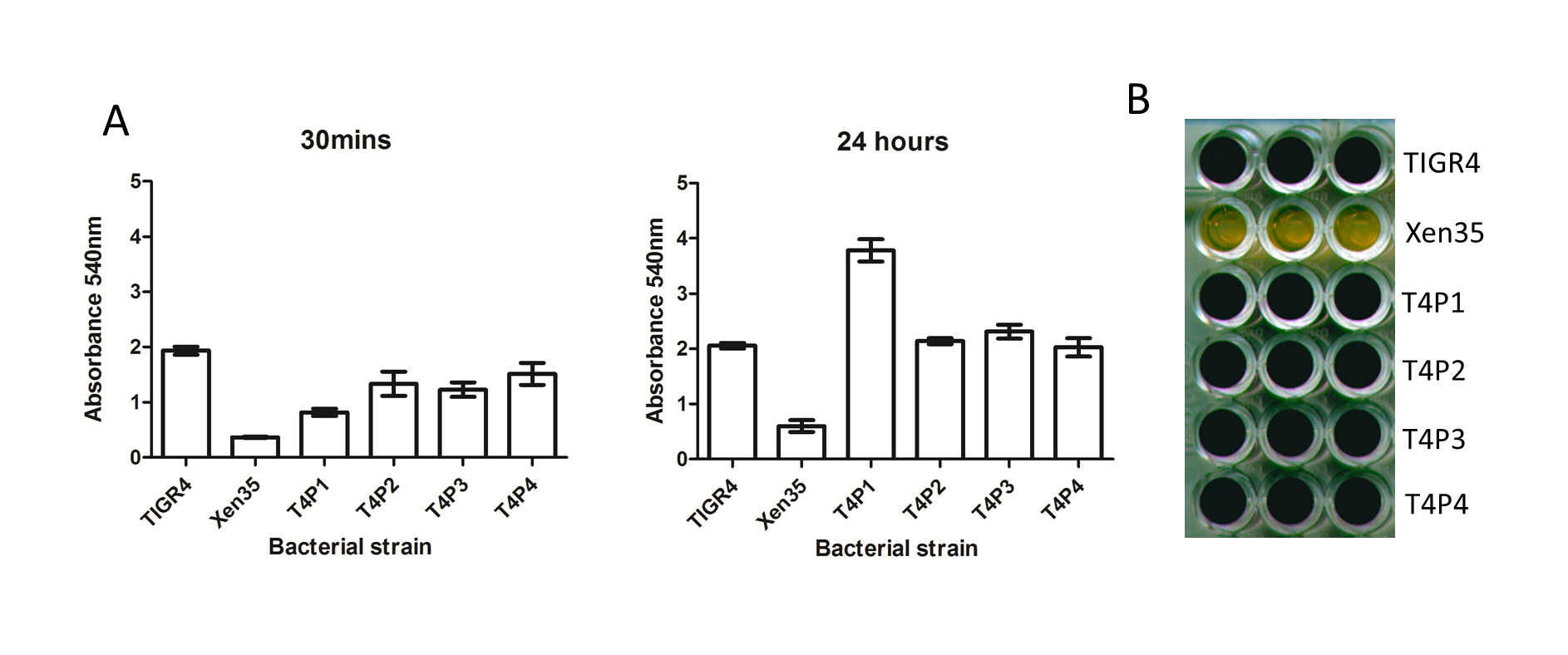

Supplement: S4 Fig — Each strain was represented in triplicate in the Hydrogen peroxide assay. (A) The graph gives the absorbance at 540nm at 30 minutes and 24 hours post incubation with the chromogenic substrate (B) Shows visually the assay performed in a 96 well plate at 24 hours post incubation. (TIF) [file pone.0189426.s004.tif]

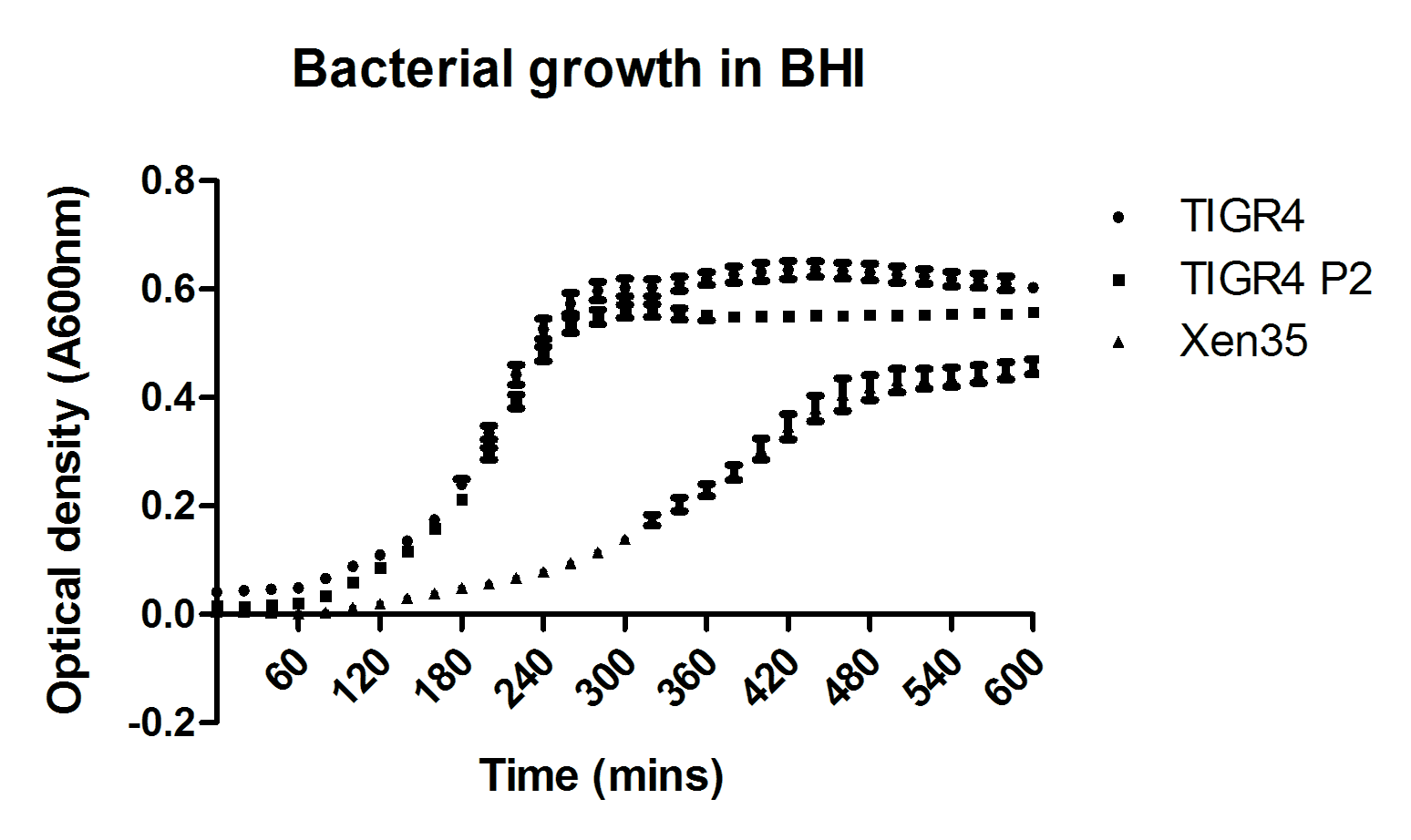

Supplement: S5 Fig — Graph of growth rates of TIGR4, Xen35 and T4P strains over time when grown in BHI broth. Each point on the graph represents the average of a triplicate experiments. Absorbance readings (600nm) were taken every 20 minutes for 10 hours. (TIF) [file pone.0189426.s005.tif]

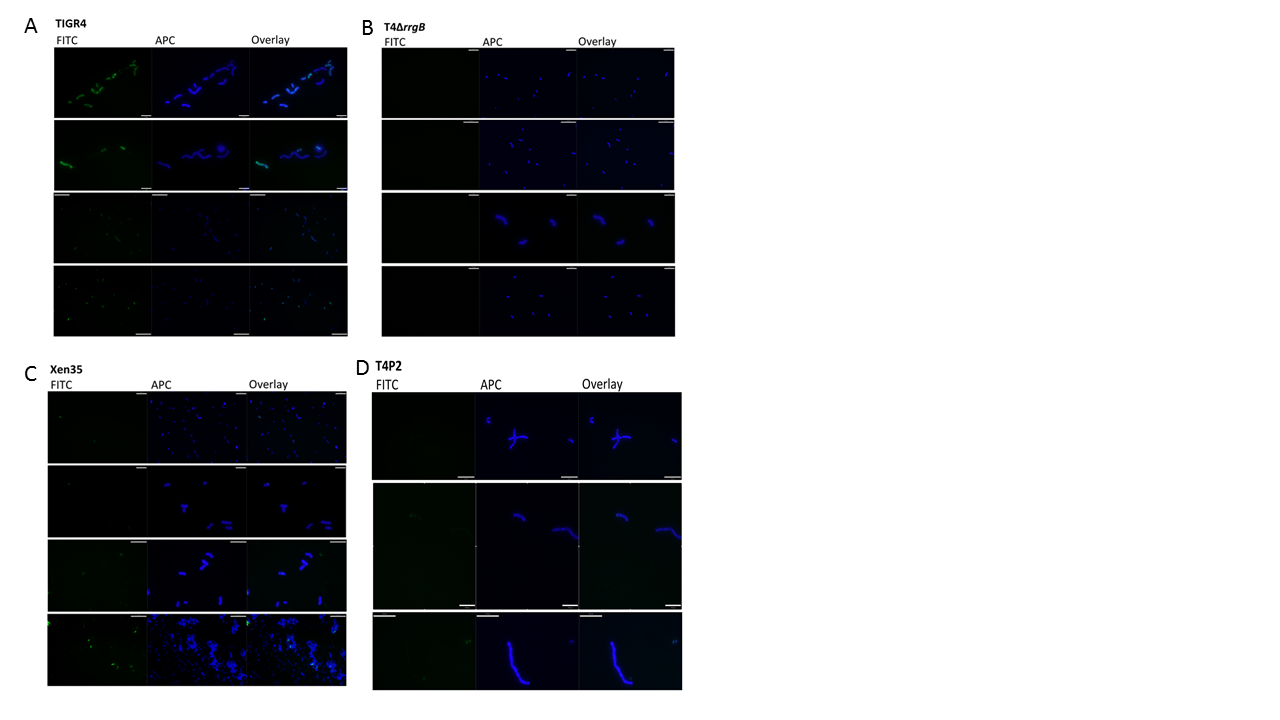

Supplement: S6 Fig — Three to four representative image of fluorescently labelled TIGR4 (A), T4ΔrrgB (B), Xen35 (C) and T4P2 (D) used for FACS analysis. Cells were stained for the presence of RrgB (FITC) and the capsule (APC). All fluorescence microscopy images were taken at X40 and X100 magnification using a Zeiss AxioscopeM1 fluorescence microscope. (TIF) [file pone.0189426.s006.tif]
