# Supplementary material for: Expression of the lux genes in Streptococcus pneumoniae modulates pilus expression and virulence
Source: PLoS One. 2018 Jan 17;13(1):e0189426. doi: 10.1371/journal.pone.0189426 (PMC5771582; doi:10.1371/journal.pone.0189426)
Supplement: S1 Table — Table lists all 243 genome changes in Xen35 compared to TIGR4. Information includes the type of change with three types of SNP, SNP-NS (NON SYNONYMOUSE SNP), SNP-S (SYNONYMOUSE SNP) and SNP-D (Dynamic SNP, mix of both). Position of change is included in the TIGR4 genome sequence data and the Xen35 genome sequence data. Changes are also stated whether it correspond to that of D39 or neither (not in D39 or TIGR4). (DOCX) [file pone.0189426.s007.docx]

Table S1: Names of all *S.pneumoniae* strains used in this study.

Table of all *S.pneumoniae* strains used in this study with their antibiotic profiles. All strains unless a reference is given were constructed by the author.

| Strain name | Description | Reference |
| --- | --- | --- |
| TIGR4 | Serotype 4, TIGR4 strain | (Aaberge *et al.*, 1995; Tettelin *et al.*, 2001) |
| Xen35 | Serotype 4 TIGR4 strain containing *luxA-E* in SP_1914. | (Francis *et al.*, 2001; Orihuela *et al.*, 2003) |
| T4P1 | Serotype 4 TIGR4 strain with *lux* genes inserted in SP_1886 under the control of promoter P1. | This study |
| T4P2 | Serotype 4 TIGR4 strain with *lux* genes inserted in SP_1886 under the control of promoter P2. | This study |
| T4P3 | Serotype 4 TIGR4 strain with *lux* genes inserted in SP_1886 under the control of promoter P3. | This study |
| T4P4 | Serotype 4 TIGR4 strain with *lux* genes inserted in SP_1886 under the control of promoter P4. | This study |
| pCEP2 | Chromosomal expression platform | (Guiral *et al.*, 2006) |

Aaberge, I.S.., J. Engz., G. Lermark & M. Levik, (1995) Virulence of Streptococcuspneumoniaein mice: a standardized method for preparation and frozen storage of the experimental bacterial inoculum. *Microbial pathogenesis*: 141-152.

Francis, K.P.., J. Yu., C. Bellinger-Kawahara., D. Joh., M.J. Hawkinson., G. Xiao*, et al.*, (2001) Visualizing pneumococcal infections in the lungs of live mice using bioluminescent Streptococcus pneumoniae transformed with a novel gram-positive lux transposon. *Infection and immunity* **69**: 3350-3358.

Guiral, S.., V. Henard., M.H. Laaberki., C. Granadel., M. Prudhomme., B. Martin & J.P. Claverys, (2006) Construction and evaluation of a chromosomal expression platform (CEP) for ectopic, maltose-driven gene expression in Streptococcus pneumoniae. *Microbiology* **152**: 343-349.

Orihuela, C.J.., G. Gao., M. McGee., J. Yu., K.P. Francis & E. Tuomanen, (2003) Organ-specific models of Streptococcus pneumoniae Disease. *Scandinavian Journal of Infectious Diseases* **35**: 647-652.

Tettelin, H.., K.E. Nelson., I.T. Paulsen., J.A. Eisen., T.D. Read., S. Peterson*, et al.*, (2001) Complete genome sequence of a virulent isolate of Streptococcus pneumoniae. *Science* **293**: 498-506.
