# Supplementary material for: Expression of the lux genes in Streptococcus pneumoniae modulates pilus expression and virulence
Source: PLoS One. 2018 Jan 17;13(1):e0189426. doi: 10.1371/journal.pone.0189426 (PMC5771582; doi:10.1371/journal.pone.0189426)
Supplement: S2 Table — RNA-seq gene expression changes observed in Xen35 compared to TIGR4. Table shows list of significantly differentially regulated genes with over 2 fold change. All genes were found to be differentially regulated when data was aligned to TIGR4 and Xen35 independently, With the exception of SP_1915 and SP_0517, which were only differentially regulated when data was aligned to TIGR4. Fold change represent the average fold change between the two analyses bar the two genes noted above. (DOCX) [file pone.0189426.s008.docx]

Table S2: Primers and related information used for RT-PCR analysis.

| **Gene** | **Gene number** | **Primer name** | **DNA sequence 5’-3’** | **Size (bp)** |
| --- | --- | --- | --- | --- |
| *rlrA* | SP_0461 | 0461 RT-F | CCATCGCAACAGGCTACC | 185 |
|  |  | 0461 RT-R | TGTGACCCAATCCATACTTCC |  |
| *rrgA* | SP_0462 | 0462 RT-F | AACCAGTCCAGCGATAGG | 185 |
|  |  | 0462 RT-R | CTTCTGTCAAGGTGTATGTCC |  |
| *rrgB* | SP_0463 | 0463 RT-F | ATACACCTGTGAACCACCAAG | 104 |
|  |  | 0463 RT-R | CATTCTATCGCTCCAGTTTGC |  |
| *rrgC* | SP_0464 | 0464 RT-F | GTATCTTCTTTGTTATGGCTCTG | 185 |
|  |  | 0464 RT-R | ATCATCATAGGAATACGAATCATC |  |
| *srtB* | SP_0466 | 0466 RT-F | GGTGTCTCGCTTGTATTATCG | 86 |
|  |  | 0466 RT-R | TGTCAGCCTCATCCAACG |  |
| *srtC* | SP_0467 | 0467 RT-F | GTGTCTCGTTATTATTATCGTATTG | 91 |
|  |  | 0467 RT-R | CCTCAAGTTCTGCCTTATCC |  |
| *srtD* | SP_0468 | 0468 RT-F | TCTCGCCTACAATCAACGC | 169 |
|  |  | 0468 RT-R | ATAATCTGCTCCCAAATAAACCG |  |
| *gyrA* | SP_1219 | gyrA RT-F | GCGCGAGCTCTTCCTGATGT | 100 |
|  |  | gyrA RT-R | TATGGGGTTTGTCTGGGGTC |  |
| *psrP* | SP_1772 | PsrP RT-F | AATGAGTCAGCAGTACTTG | 100 |
|  |  | PsrP RT-R | TCGCTGAATTACTTGTAG |  |
| Hypothetical | SP_1914 | 1914 RT-F | TGGCGTGTAGATTTGAAAGTAG | 127 |
|  |  | 1914 RT-R | CGTAGACAATTTCCAACAACC |  |
| Hypothetical | SP_1915 | RT-1915N*-F | TCAAGCTCTATGAGTTGGAGTC | 118 |
|  |  | 1915 RT-R | TTAGGAGGGATTGGTAATGCCG |  |
| *luxA* |  | LuxA RT-F | GGAGCATCATTTCACGGAGTTTG | 114 |
|  |  | LuxA RT-R | GTGGGAAGAACAATAGCGGCAGT |  |
| *luxB* |  | LuxB RT-F | CAGATAATGGTGTTGTCGGCG | 120 |
|  |  | LuxB RT-RN | CTATGCGGACAGGA TGA TGAGTTG |  |
| *luxC* |  | LuxC RT-F | GTGTTTACCTGCCAATATTGAATGAC | 166 |
|  |  | LuxC RT-R | TTTAAGTCACGAATGTATGTCCTGCG |  |
| *luxD* |  | LuxD RT-F | GCCAGAAGAAAACAGCCCAAAGAG | 88 |
|  |  | LuxD RT-R | CGCCAGACCAGCAAAATGAT |  |
| *luxE* |  | LuxE RT-F | TGATGATTTGATTTTTTCGAGCG | 161 |
|  |  | LuxE RT-R | CCGTAATATTGTCATCTACTTTGTGTGC |  |
