# Supplementary material for: Expression of the lux genes in Streptococcus pneumoniae modulates pilus expression and virulence
Source: PLoS One. 2018 Jan 17;13(1):e0189426. doi: 10.1371/journal.pone.0189426 (PMC5771582; doi:10.1371/journal.pone.0189426)
Supplement: S3 Table — (DOCX) [file pone.0189426.s009.docx]

Table S3: Table of primers used for construction of T4P strains

| **Gene** | **Primer name** | **DNA sequence 5’-3’** | **size (bp)** |
| --- | --- | --- | --- |
| *lux* genes | PcepluxF | TGCTACCATGGAATTTGGAAACTTTTTGC | 5606 |
|  | PcepluxR | CAAACGGATCCTTAACTATCAAACGCTTCGGTT |  |
| SDM | Puc18 LSD F | GTTCCGAAAACCAAAGGC**C**TTGCGCTTGATAAGTTTG | 12,431 |
|  | Puc18 LSD R | CAAGGCTTTTGGTTTCCG**G**AACGCGAACTATTCAAAC |  |
| 5’ SP_1489 | 5’ SP_1489F | GCTAGGCCTGTGGATGGATAATGCTGA | 206 |
|  | 5’ SP_1489R | TAGCCATGGTTGAGTAAAAGCCTCCAAT |  |
| 5’ SP_2012 | 5’ SP_2012F | CTAGGCCTTAAGGGATTCCTTGGTTTAC | 176 |
|  | 5’ SP_2012R | TAGCCATGGT AGTGA TTTCCTCCTTA TG |  |
| 5’ SP_1128 | 5’ SP_1128F | GCTAGGCCTGAAAACAGTATATCATAAA | 163 |
|  | 5’ SP_1128R | TAGCCATGGTTTTTACTCTCCTTATGAG |  |
| 5’ SP_0232 | 5’ SP_0232F | TAGGCCTAGCGTTTTTCACACTTGC | 116 |
|  | 5’ SP_0232R | GCCATGGGCAAAAGCACCTCCATAA |  |
| 5’ SP_1915 | 5’ SP_1915F | TAGGCCTCATGTAAGTTAAGCTAGTC | 140 |
|  | 5’ SP_1915R | GCCATGGATCTCCTTTCTGACTCTA |  |
