# Supplementary material for: Expression of the lux genes in Streptococcus pneumoniae modulates pilus expression and virulence
Source: PLoS One. 2018 Jan 17;13(1):e0189426. doi: 10.1371/journal.pone.0189426 (PMC5771582; doi:10.1371/journal.pone.0189426)
Supplement: S4 Table — Table of all S. pneumoniae strains used in this study with their antibiotic profiles. All strains unless a reference is given were constructed by the author. (DOCX) [file pone.0189426.s010.docx]

Table S4: Table of promoters used to drive expression of the *lux* genes

| **Promoter** | **Gene number** | **Gene function** | **RPKM expression value** | **Promoter 5’ of gene** |
| --- | --- | --- | --- | --- |
| P1 | SP_1489 | Elongation factor Tu | 8,057.38 | SP_1489 |
| P2 | SP_2012 | GAPDH | 5,126.58 | SP_2012 |
| P3 | SP_1128 | Enolase | 3,202.48 | SP_1128 |
| P4 | SP_0236 | DNA directed RNA polymerase alpha | 3,178.91 | SP_0232 |
| P19 | SP_1915 | Hypothetical protein | 34.84 | SP_1915 |
