# Supplementary material for: Expression of the lux genes in Streptococcus pneumoniae modulates pilus expression and virulence
Source: PLoS One. 2018 Jan 17;13(1):e0189426. doi: 10.1371/journal.pone.0189426 (PMC5771582; doi:10.1371/journal.pone.0189426)
Supplement: S5 Table — (DOCX) [file pone.0189426.s011.docx]

Table S5: Whole genome change in Xen35 compared to TIGR4

Table lists all 243 genome changes in Xen35 compared to TIGR4. Information includes the type of change with three types of SNP, SNP-NS (NON SYNONYMOUSE SNP), SNP-S (SYNONYMOUSE SNP) and SNP-D (Dynamic SNP, mix of both). Position of change is included in the TIGR4 genome sequence data and the Xen35 genome sequence data. Changes are also stated whether it correspond to that of D39 or neither (not in D39 or TIGR4).

|  | Gene | Change Type | Change | Position TIGR4 | Position Xen35 | Origin (D39/TIGR4/Neither) |
| --- | --- | --- | --- | --- | --- | --- |
| 1 | SP_0148 | SNP-NS | G>C | 146055 | 146055 | D39 |
| 2 | SP_0200/201 | DEL | C | 188293 | 188292-188293 | D39 |
| 3 | SP_0206 | INS | C | 192436-192437 | 192435 | D39 |
| 4 | SP_0272 | SNP-NS | A>G | 247805 | 247805 | D39 |
| 5 | INT 0296-0297 | DEL | G | 273379 | 273378-273379 | Neither |
| 6 | INT 0482-0483 | SNP | G>A | 463630 | 463629 | D39 |
| 7 | INT 0482-0483 | SNP | A>G | 463631 | 463630 | D39 |
| 8 | SP_0491 | DEL | C | 469288 | 469286-469287 | D39 |
| 9 | INT 0496-0497 | SNP | G>T | 476406 | 476404 | D39 |
| 10 | INT 0630-0631 | INS | G | 597325-597326 | 597324 | D39 |
| 11 | SP_0715 | SNP-NS | G>T | 681177 | 681176 | Neither |
| 12 | SP_0730 | SNP-NS | G>T | 695462 | 695461 | Neither |
| 13 | INT 0771-0772 | SNP | C>T | 730678 | 730677 | D39 |
| 14 | SP-0784 | SNP-NS | A>G | 737108 | 737107 | D39 |
| 15 | SP_0807 | SNP-NS | C>A | 762843 | 762842 | Neither |
| 16 | INT 0885-0885 | INS | C | 834923-834924 | 834923 | D39 |
| 17 | SP_0904 | SNP-D | T>G (48.5/ 51.5) | 857447 | 857447 | Neither |
| 18 | SP_0927 | SNP-D | C>T (42.1/ 57.9) | 880600 | 880600 | Neither |
| 19 | INT 1029-1030 | DEL | T | 972510 | 972509-972510 | D39 |
| 20 | INT 1053-1054 | INS | C | 991106-991107 | 991106 | D39 |
| 21 | SP_1166 | SNP-NS | C>G | 1101585 | 1101585 | D39 |
| 22 | SP_1166 | SNP-NS | G>C | 1101586 | 1101586 | D39 |
| 23 | INT 1175-1176 | INS | C | 1113285-1113286 | 1113286 | D39 |
| 24 | SP_1190 | SNP-NS | C>G | 1127020 | 1127022 | D39 |
| 25 | SP_1190 | SNP-NS | G>C | 1127021 | 1127023 | D39 |
| 26 | INT 1199-1200 | INS | G | 1132344-1132345 | 1132346 | D39 |
| 27 | INT 1199-1200 | INS | G | 1132391-1132392 | 1132394 | Neither |
| 28 | INT 1199-1200 | SNP | G>A | 1132435 | 1132438 | D39 |
| 29 | INT 1199-1200 | SNP | A>G | 1132436 | 1132439 | D39 |
| 30 | SP_1343 | SNP-NS | C>G | 1267219 | 1267222 | D39 |
| 31 | SP_1343 | SNP-NS | G>C | 1267220 | 1267223 | D39 |
| 32 | SP_1631 | SNP-S | C>A | 1530636 | 1530639 | Neither |
| 33 | SP_1702 | SNP-S | G>T | 1608798 | 1608801 | Neither |
| 34 | SP_1715 | SNP-NS | A>G | 1618223 | 1618226 | D39 |
| 35 | SP_1715 | INS | G | 1618515-1618516 | 1618519 | D39 |
| 36 | INT 1717-1718 | SNP | G>A | 1622021 | 1622025 | D39 |
| 37 | INT 1717-1718 | SNP | A>G | 1622022 | 1622026 | D39 |
| 38 | INT 1717-1718 | SNP | G>A | 1622066 | 1622070 | D39 |
| 39 | INT 1717-1718 | SNP | A>G | 1622067 | 1622071 | D39 |
| 40 | SP_1732 | DEL | 216bp | 1634948-1635163 | 1634951-1634952 | Neither |
| 41 | SP_1732 | SNP-S | A>G | 1635179 | 1634967 | Neither |
| 42 | SP_1733 | SNP-NS | G>C | 1636745 | 1636533 | Neither |
| 43 | INT 1776-1777 | INS | T | 1695199-1695200 | 1694988 | Neither |
| 44 | INT 1777-1778 | INS | C | 1696085-1696086 | 1695875 | D39 |
| 45 | INT 1851-1852 | DEL | A | 1759031 | 1758820-1758821 | D39 |
| 46 | SP_1891 | SNP-NS | G>A | 1796273 | 1796062 | Neither |
| 47 | SP_1908 | SNP-S | G>C | 1822548 | 1822337 | D39 |
| 48 | SP_1908 | SNP-S | T>G | 1822554 | 1822343 | D39 |
| 49 | SP_1908 | SNP-S | C>T | 1822563 | 1822352 | D39 |
| 50 | SP_1908 | SNP-S | G>A | 1822566 | 1822355 | D39 |
| 51 | SP_1908 | SNP-S | G>A | 1822569 | 1822358 | D39 |
| 52 | SP_1908 | SNP-S | T>C | 1822575 | 1822364 | D39 |
| 53 | SP_1908 | SNP-S | G>A | 1822578 | 1822367 | D39 |
| 54 | SP_1908 | SNP-S | T>C | 1822668 | 1822457 | Neither |
| 55 | SP_1908 | SNP-NS | G>A | 1822699 | 1822488 | Neither |
| 56 | SP_1908 | SNP-S | C>G | 1822737 | 1822526 | D39 |
| 57 | SP_1908 | SNP-S | C>T | 1822761 | 1822550 | Neither |
| 58 | SP_1908 | SNP-S | T>C | 1822809 | 1822598 | D39 |
| 59 | SP_1908 | SNP-NS | A>T | 1822817 | 1822606 | D39 |
| 60 | INT 1908-1909 | SNP | A>G | 1822905 | 1822694 | D39 |
| 61 | INT 1908-1909 | SNP | T>C | 1822912 | 1822701 | D39 |
| 62 | SP_1909 | SNP-S | A>T | 1823414 | 1823203 | D39 |
| 63 | SP_1909 | SNP-S | G>A | 1823422 | 1823211 | D39 |
| 64 | SP_1909 | SNP-S | G>A | 1823426 | 1823215 | D39 |
| 65 | SP_1909 | SNP-NS | T>C | 1823433 | 1823222 | D39 |
| 66 | SP_1909 | SNP-NS | G>T | 1823434 | 1823223 | D39 |
| 67 | SP_1909 | SNP-S | A>C | 1823435 | 1823224 | D39 |
| 68 | SP_1910 | SNP-S | T>C | 1823797 | 1823586 | D39 |
| 69 | SP_1910 | SNP-S | G>A | 1823811 | 1823600 | D39 |
| 70 | SP_1910 | SNP-S | G>A | 1823830 | 1823619 | D39 |
| 71 | SP_1910 | SNP-S | A>C | 1823839 | 1823628 | D39 |
| 72 | SP_1910 | SNP-S | A>G | 1823866 | 1823655 | D39 |
| 73 | SP_1910 | SNP-NS | G>A | 1823880 | 1823669 | D39 |
| 74 | SP_1910 | SNP-S | A>G | 1823899 | 1823688 | D39 |
| 75 | SP_1910 | SNP-S | T>A | 1823902 | 1823691 | D39 |
| 76 | SP_1910 | SNP-S | C>A | 1823911 | 1823700 | D39 |
| 77 | SP_1910 | SNP-S | G>C | 1823914 | 1823703 | D39 |
| 78 | SP_1910 | SNP-S | C>A | 1823932 | 1823721 | D39 |
| 79 | SP_1910 | SNP-S | G>A | 1823944 | 1823733 | D39 |
| 80 | SP_1910 | SNP-S | A>T | 1823947 | 1823736 | D39 |
| 81 | SP_1910 | SNP-S | T>C | 1823965 | 1823754 | D39 |
| 82 | SP_1910 | SNP-NS | A>T | 1823968 | 1823757 | D39 |
| 83 | SP_1910 | SNP-NS | G>A | 1823969 | 1823758 | D39 |
| 84 | SP_1910 | SNP-S | A>G | 1823974 | 1823763 | D39 |
| 85 | SP_1910 | SNP-S | A>G | 1824016 | 1823805 | D39 |
| 86 | SP_1910 | SNP-S | G>A | 1824025 | 1823814 | D39 |
| 87 | SP_1910 | SNP-S | A>G | 1824037 | 1823826 | D39 |
| 88 | SP_1910 | SNP-S | T>A | 1824049 | 1823838 | D39 |
| 89 | SP_1910 | SNP-S | C>T | 1824106 | 1823895 | D39 |
| 90 | SP_1910 | SNP-S | C>T | 1824109 | 1823898 | D39 |
| 91 | SP_1910 | SNP-NS | T>A | 1824141 | 1823930 | D39 |
| 92 | SP_1910 | SNP-S | C>T | 1824142 | 1823931 | D39 |
| 93 | SP_1910 | SNP-S | T>C | 1824175 | 1823964 | D39 |
| 94 | SP_1910 | SNP-S | A>G | 1824178 | 1823967 | D39 |
| 95 | SP_1910 | SNP-S | T>C | 1824181 | 1823970 | D39 |
| 96 | SP_1910 | SNP-S | T>C | 1824187 | 1823976 | D39 |
| 97 | SP_1910 | SNP-S | G>A | 1824190 | 1823979 | D39 |
| 98 | SP_1910 | SNP-S | A>G | 1824196 | 1823985 | D39 |
| 99 | SP_1910 | SNP-S | C>T | 1824205 | 1823994 | D39 |
| 100 | SP_1910 | SNP-NS | A>T | 1824220 | 1824009 | D39 |
| 101 | SP_1910 | SNP-S | A>G | 1824229 | 1824018 | D39 |
| 102 | SP_1910 | SNP-S | G>A | 1824253 | 1824042 | D39 |
| 103 | SP_1910 | SNP-S | C>T | 1824256 | 1824045 | D39 |
| 104 | SP_1910 | SNP-NS | C>T | 1824264 | 1824053 | D39 |
| 105 | SP_1910 | SNP-S | A>G | 1824325 | 1824114 | D39 |
| 106 | SP_1910 | SNP-S | T>C | 1824331 | 1824120 | D39 |
| 107 | SP_1911 | SNP-S | G>A | 1824366 | 1824155 | D39 |
| 108 | SP_1911 | SNP-S | G>A | 1824379 | 1824168 | D39 |
| 109 | SP_1911 | SNP-S | A>G | 1824382 | 1824171 | D39 |
| 110 | SP_1911 | SNP-S | G>A | 1824385 | 1824174 | D39 |
| 111 | SP_1911 | SNP-NS | T>G | 1824391 | 1824180 | Neither |
| 112 | SP_1911 | SNP-NS | G>C | 1824393 | 1824182 | D39 |
| 113 | SP_1911 | SNP-S | G>A | 1824397 | 1824186 | D39 |
| 114 | SP_1911 | SNP-S | C>T | 1824547 | 1824336 | D39 |
| 115 | SP_1911 | SNP-NS | T>C | 1824588 | 1824377 | D39 |
| 116 | SP_1911 | SNP-NS | T>G | 1824609 | 1824398 | D39 |
| 117 | SP_1912 | SNP-S | G>A | 1824732 | 1824521 | D39 |
| 118 | SP_1912 | SNP-S | A>G | 1824795 | 1824584 | D39 |
| 119 | SP_1912 | SNP-S | A>G | 1824897 | 1824686 | D39 |
| 120 | INT 1912-1913 | SNP | G>A | 1825207 | 1824996 | D39 |
| 121 | SP_1913 | SNP-NS | T>C | 1825256 | 1825045 | D39 |
| 122 | SP_1914 | SNP-NS | A>G | 1825712 | 1825501 | D39 |
| 123 | SP_1914 | SNP-S | C>T | 1825822 | 1825611 | D39 |
| 124 | SP_1914 | SNP-NS | G>T | 1826142 | 1825931 | D39 |
| 125 | SP_1915 | SNP-S | G>C | 1826391 | 1834074 | D39 |
| 126 | SP_1915 | SNP-S | C>G | 1826409 | 1834092 | D39 |
| 127 | SP_1915 | SNP-S | C>T | 1826412 | 1834095 | D39 |
| 128 | SP_1915 | SNP-S | T>C | 1826457 | 1834140 | D39 |
| 129 | SP_1915 | SNP-S | G>A | 1826460 | 1834143 | D39 |
| 130 | SP_1915 | SNP-S | A>G | 1826463 | 1834146 | D39 |
| 131 | SP_1915 | SNP-S | C>T | 1826466 | 1834149 | D39 |
| 132 | SP_1915 | SNP-S | A>C | 1826469 | 1834152 | D39 |
| 133 | SP_1915 | SNP-S | G>A | 1826472 | 1834155 | D39 |
| 134 | SP_1915 | SNP-S | G>A | 1826475 | 1834158 | D39 |
| 135 | SP_1915 | SNP-NS | T>A | 1826478 | 1834161 | D39 |
| 136 | SP_1915 | SNP-NS | G>A | 1826479 | 1834162 | D39 |
| 137 | SP_1915 | SNP-S | A>T | 1826496 | 1834179 | D39 |
| 138 | SP_1915 | SNP-NS | T>C | 1826503 | 1834186 | D39 |
| 139 | SP_1915 | SNP-S | C>A | 1826517 | 1834200 | D39 |
| 140 | SP_1915 | SNP-S | A>G | 1826519 | 1834202 | D39 |
| 141 | SP_1915 | SNP-NS | A>C | 1826525 | 1834208 | D39 |
| 142 | SP_1915 | SNP-S | G>C | 1826691 | 1834374 | D39 |
| 143 | SP_1916 | SNP-NS | C>T | 1827142 | 1834825 | D39 |
| 144 | SP_1916 | SNP-NS | A>G | 1827352 | 1835035 | D39 |
| 145 | SP_1916 | SNP-NS | G>T | 1827368 | 1835051 | D39 |
| 146 | SP_1917 | SNP-S | T>G | 1827582 | 1835265 | D39 |
| 147 | SP_1917 | SNP-S | A>G | 1827628 | 1835311 | D39 |
| 148 | INT 1917-1918 | SNP | C>T | 1827808 | 1835491 | D39 |
| 149 | SP_1918 | SNP-NS | G>A | 1828094 | 1835777 | D39 |
| 150 | SP_1918 | SNP-NS | G>A | 1828115 | 1835798 | D39 |
| 151 | SP_1918 | SNP-NS | T>C | 1828142 | 1835825 | D39 |
| 152 | SP_1919 | SNP-NS | G>A | 1828907 | 1836590 | D39 |
| 153 | SP_1919 | SNP-NS | C>T | 1829040 | 1836723 | D39 |
| 154 | SP_1919 | SNP-NS | A>T | 1829041 | 1836724 | D39 |
| 155 | SP_1919 | SNP-NS | T>C | 1829231 | 1836914 | D39 |
| 156 | SP_1919 | SNP-NS | A>G | 1829312 | 1836995 | D39 |
| 157 | SP_1919 | SNP-NS | G>A | 1829318 | 1837001 | D39 |
| 158 | SP_1919 | SNP-NS | C>T | 1829335 | 1837018 | D39 |
| 159 | SP_1919 | SNP-NS | G>A | 1829383 | 1837066 | D39 |
| 160 | SP_1919 | SNP-NS | A>G | 1829537 | 1837220 | D39 |
| 161 | SP_1919 | SNP-NS | A>G | 1829561 | 1837244 | D39 |
| 162 | SP_1919 | SNP-NS | T>C | 1829587 | 1837270 | D39 |
| 163 | SP_1920 | DEL | T | 1829756 | 1837438-1837439 | D39 |
| 164 | SP_1920 | SNP | C>T | 1829777 | 1837459 | D39 |
| 165 | SP_1920 | DEL | A | 1829840 | 1837521-1837522 | D39 |
| 166 | SP_1920 | SNP-NS | G>A | 1829980 | 1837661 | D39 |
| 167 | SP_1920 | SNP-NS | G>A | 1830130 | 1837811 | D39 |
| 168 | SP_1922 | SNP-S | G>A | 1830467 | 1838148 | D39 |
| 169 | SP_1922 | SNP-S | G>A | 1830626 | 1838307 | D39 |
| 170 | SP_1922 | SNP-S | G>A | 1830707 | 1838388 | D39 |
| 171 | SP_1922 | SNP-S | A>G | 1830962 | 1838643 | D39 |
| 172 | INT 1922-1923 | SNP | G>A | 1831680 | 1839361 | D39 |
| 173 | INT 1922-1923 | SNP | T>C | 1831772 | 1839453 | D39 |
| 174 | INT 1922-1923 | SNP | T>C | 1831785 | 1839466 | D39 |
| 175 | SP_1923 | SNP-S | T>C | 1831917 | 1839598 | D39 |
| 176 | SP_1923 | SNP-S | T>C | 1832100 | 1839781 | D39 |
| 177 | SP_1923 | SNP-NS | T>C | 1832174 | 1839855 | D39 |
| 178 | SP_1923 | SNP-S | A>G | 1832274 | 1839955 | D39 |
| 179 | SP_1923 | SNP-S | T>C | 1832475 | 1840156 | D39 |
| 180 | SP_1923 | SNP-S | A>G | 1832697 | 1840378 | D39 |
| 181 | SP_1923 | SNP-S | G>A | 1832754 | 1840435 | D39 |
| 182 | SP_1923 | SNP-S | G>T | 1832853 | 1840534 | D39 |
| 183 | SP_1923 | SNP-S | G>A | 1833036 | 1840717 | D39 |
| 184 | SP_1924 | SNP-NS | A>G | 1833393 | 1841074 | D39 |
| 185 | SP_1924 | SNP-S | A>G | 1833447 | 1841128 | D39 |
| 186 | SP_1924 | SNP-S | G>A | 1833463 | 1841144 | D39 |
| 187 | SP_1925 | SNP-S | G>A | 1834319 | 1842000 | D39 |
| 188 | INT 1926-1927 | SNP | T>C | 1834835 | 1842516 | D39 |
| 189 | INT 1926-1927 | SNP | T>C | 1834897 | 1842578 | D39 |
| 190 | SP_1927 | SNP-NS | T>G | 1834935 | 1842616 | D39 |
| 191 | SP_1927 | SNP-S | C>T | 1834953 | 1842634 | Neither |
| 192 | SP_1927 | SNP-NS | G>A | 1834962 | 1842643 | Neither |
| 193 | SP_1927 | SNP-S | C>T | 1834994 | 1842675 | Neither |
| 194 | SP_1927 | SNP-S | G>C | 1834997 | 1842678 | Neither |
| 195 | SP_1927 | SNP-NS | A>G | 1835012 | 1842693 | D39 |
| 196 | SP_1928 | SNP-NS | G>A | 1835482 | 1843163 | Neither |
| 197 | SP_1928 | SNP-NS | G>A | 1835483 | 1843164 | Neither |
| 198 | SP_1928 | SNP-NS | G>A | 1835507 | 1843188 | D39 |
| 199 | SP_1928 | SNP-S | G>A | 1835550 | 1843231 | Neither |
| 200 | SP_1928 | SNP-NS | C>T | 1835558 | 1843239 | Neither |
| 201 | SP_1928 | SNP-S | C>T | 1835571 | 1843252 | Neither |
| 202 | SP_1928 | SNP-NS | G>A | 1835629 | 1843310 | D39 |
| 203 | SP_1928 | SNP-NS | A>G | 1835657 | 1843338 | Neither |
| 204 | SP_1928 | SNP-S | T>C | 1835679 | 1843360 | D39 |
| 205 | SP_1928 | SNP-NS | T>G | 1835699 | 1843380 | D39 |
| 206 | INT 1928-1929 | INS | AT | 1835757-1835758 | 1843439-1843440 | D39 |
| 207 | INT 1928-1929 | INS | 24bp | 1835782-1835783 | 1843466-1843489 | D39 |
| 208 | INT 1928-1929 | SNP | T>C | 1835785 | 1843492 | D39 |
| 209 | INT 1928-1929 | INS | A | 1835840-1835841 | 1843548 | D39 |
| 210 | INT 1928-1929 | SNP | C>A | 1835887 | 1843595 | D39 |
| 211 | INT 1928-1929 | SNP | C>T | 1835906 | 1843614 | D39 |
| 212 | INT 1928-1929 | SNP | G>T | 1835981 | 1843689 | D39 |
| 213 | INT 1928-1929 | SNP | T>C | 1835996 | 1843704 | D39 |
| 214 | INT 1928-1929 | SNP | A>G | 1836026 | 1843734 | D39 |
| 215 | INT 1929-1930 | SNP | C>T | 1836244 | 1843952 | D39 |
| 216 | INT 1929-1930 | SNP | G>A | 1836297 | 1844005 | D39 |
| 217 | INT 1929-1930 | SNP | A>G | 1836431 | 1844139 | D39 |
| 218 | SP_1930 | SNP-NS | G>A | 1836482 | 1844190 | D39 |
| 219 | SP_1931 | SNP-S | C>T | 1836802 | 1844510 | D39 |
| 220 | SP_1931 | SNP-S | A>G | 1836879 | 1844587 | D39 |
| 221 | SP_1931 | SNP-NS | T>A | 1836923 | 1844631 | D39 |
| 222 | SP_1931 | SNP-NS | G>A | 1836993 | 1844701 | D39 |
| 223 | SP_1931 | SNP-NS | G>A | 1837074 | 1844782 | D39 |
| 224 | SP_1933 | SNP-NS | A>C | 1837534 | 1845242 | Neither |
| 225 | SP_1933 | SNP-NS | C>T | 1837543 | 1845251 | Neither |
| 226 | SP_1933 | SNP-NS | A>G | 1837602 | 1845310 | Neither |
| 227 | INT 1933-1934 | SNP | G>C | 1838416 | 1846124 | Neither |
| 228 | INT 1933-1934 | SNP | T>C | 1838432 | 1846140 | Neither |
| 229 | INT 1933-1934 | SNP | A>G | 1838721 | 1846429 | D39 |
| 230 | SP_1934 | SNP-NS | A>G | 1838778 | 1846486 | D39 |
| 231 | SP_1934 | SNP-S | T>A | 1838869 | 1846577 | D39 |
| 232 | SP_1934 | SNP-NS | C>G | 1838873 | 1846581 | D39 |
| 233 | SP_1934 | SNP-NS | A>G | 1839074 | 1846782 | D39 |
| 234 | SP_1934 | SNP-S | G>A | 1839103 | 1846811 | Neither |
| 235 | INT 1934-1935 | SNP | C>A | 1839182 | 1846890 | Neither |
| 236 | INT 1934-1935 | SNP | T>C | 1839276 | 1846984 | Neither |
| 237 | INT 1934-1935 | SNP | G>A | 1839277 | 1846985 | Neither |
| 238 | SP_1935 | SNP-NS | C>A | 1839292 | 1847000 | Neither |
| 239 | SP_1935 | SNP-NS | G>A | 1839298 | 1847006 | D39 |
| 240 | SP_1935 | SNP-S | T>C | 1839306 | 1847014 | D39 |
| 241 | SP_1980 | SNP-S | A>G | 1884908 | 1892616 | D39 |
| 242 | SP_2076 | INS | G | 1985218-1985219 | 1992927 | D39 |
| 243 | INT 2105-2106 | INS | G | 2016331-2016332 | 2024041 | D39 |
