# Supplementary material for: Expression of the lux genes in Streptococcus pneumoniae modulates pilus expression and virulence
Source: PLoS One. 2018 Jan 17;13(1):e0189426. doi: 10.1371/journal.pone.0189426 (PMC5771582; doi:10.1371/journal.pone.0189426)
Supplement: S6 Table — (DOCX) [file pone.0189426.s012.docx]

Table S6: Transcriptomic analysis of Xen35 compared to TIGR4

RNAseq gene expression changes observed in Xen35 compared to TIGR4. Table shows list of significantly differentially regulated genes with over 2 fold change. All genes were found to be differentially regulated when data was aligned to TIGR4 and Xen35 independently, With the exception of SP_1915 and SP_0517, which were only differentially regulated when data was aligned to TIGR4. Fold change represent the average fold change between the two analyses bar the two genes noted above.

| Gene | Function | Fold change in Xen35 |
| --- | --- | --- |
| Up regulated in Xen35 | | |
| SP_0418 | acpP | 10.4 |
| SP-0517 | dnaK | 2.4 |
| SP_0438 | gatC | 2.0 |
| SP_0515 | hrcA | 2.6 |
| SP_0232 | infA | 14.3 |
| SP_0220 | rplX | 2.3 |
| SP_1107 | rpmA, Ribosomal protein L27 | 2.6 |
| SP_1299 | rpmE2, Ribosomal protein L31 type B | 10.0 |
| SP_0222 | rpsN | 2.6 |
| SP_0218 | rpsQ, 30S ribosomal protein S17, involved in translation fidelity | 2.3 |
| SP_1539 | rpsR | 16.6 |
| SP_0213 | rpsS, 30S ribosomal protein S19. complexes with S13 that binds strongly to 16S rRNA | 2.9 |
| SP_1763 | secY_2 | 4.5 |
| SP_0107 | LysM domain contining protein | 4.5 |
| SP_0167 | Hypothetial protein | 6.6 |
| SP_0168 | Macrolide efflux protein, putative | 5.8 |
| SP_0275 | Hypothetial protein | 4.0 |
| SP_0299 | Transposase authentic frameshift | 3.1 |
| SP_0338 | ATP dependant clp protease | 2.6 |
| SP_0404 | Hypothetial protein | 3.5 |
| SP_0516 | grpE, Heat shock protein | 2.3 |
| SP_0519 | dnaJ, Chaperone protein | 4.1 |
| SP_0682 | Hypothetial protein | 5.2 |
| SP_0990 | Hypothetial protein | 2.2 |
| SP_1428 | Hypothetical protein | 10.7 |
| SP_1457 | spoU, rRNA methylase family protein | 2.4 |
| SP_1462 | Hypothetical protein. | 2.4 |
| SP_1467 | pdxT, Glutamine amidotransferase | 2.5 |
| SP_1468 | psxS, Pyridoxal biosynthesis lyase | 2.3 |
| SP_1501 | Amino acid ABC transporter, ATP binding protein | 2.1 |
| SP_1502 | Amino acid ABC transporter, permease protein | 2.4 |
| SP_1721 | scrK, fructokinase | 8.0 |
| SP_1722 | scrA, PTS system | 7.7 |
| SP_1724 | scrB, Sucrose-6-phosphate hydrolase | 12.4 |
| SP_1725 | Sucrose operon repressor | 13.7 |
| SP_1737 | DNA directed RNA polymerase subunit | 2.4 |
| SP_1757 | Hypothetical protein | 4.5 |
| SP_1758 | Glycosyl transferase | 4.7 |
| SP_1759 | secA, preprotein translocase subunit | 5.0 |
| SP_1760 | Hypothetical protein | 7.3 |
| SP_1761 | Hypothetical protein | 4.1 |
| SP_1762 | Hypothetical protein | 4.6 |
| SP_1764 | Glycosyltransferase family protein | 3.5 |
| SP_1765 | Glycosyltransferase family protein | 5.3 |
| SP_1766 | Glycosyltransferase family protein | 4.1 |
| SP_1767 | Glycosyltransferase family protein | 4.3 |
| SP_1768 | Glycosyltransferase family protein | 4.4 |
| SP_1769 | Glycosyltransferase family protein, authentic frameshift. | 4.2 |
| SP_1770 | Glycosyltransferase family protein | 5.1 |
| SP_1771 | Glycosyltransferase family protein | 4.0 |
| SP_1772 | psrP, pneumococcal serine rich repeat protein | 5.7 |
| SP_1775 | Hypothetical protein | 2.6 |
| SP_1793 | Hypothetical protein | 2.3 |
| SP_1870 | fatC, iron compound ABC transporter, permease protein | 3.4 |
| SP_1872 | fatB, iron compound ABC transporter, iron compound binding protein. | 3.2 |
| SP_1882 | Hypothetical protein | 4.1 |
| SP_1910 | Hypothetical protein | 2.2 |
| SP_1911 | Thioredoxin, putative | 2.3 |
| SP_1912 | Hypothetical protein | 9.9 |
| SP_1914 | Hypothetical protein | 60.3 |
| SP_1915 | Hypothetical protein | 215.6 |
| SP_2029 | yajC, preprotein translocase | 2.21 |
| Down regulated in Xen35 | | |
| SP_0074 | Acetyltransferase | -2.5 |
| SP_0095 | Hypothetical protein | -3.9 |
| SP_0154 | Hypothetical protein | -2.3 |
| SP_0287 | Xanthine/ uracil permease protein | -2.1 |
| SP_0461 | rlrA, transcriptional regulator | -12.6 |
| SP_0462 | rrgA, cell wall surface anchor family protein | -22.3 |
| SP_0463 | rrgB, cell wall surface anchor family protein | -14.1 |
| SP_0464 | rrgC, cell wall surface anchor family protein | -14.7 |
| SP_0466 | srtB, sortase | -9.1 |
| SP_0467 | srtC, sortase | -6.5 |
| SP_0468 | srtD, sortase | -5.4 |
| SP_0488 | Hypothetical protein | -2.6 |
| SP_0521 | HIT family protein | -2.2 |
| SP_0564 | Hypothetical protein | -3.5 |
| SP_0661 | rr09, DNA binding response regulator | -2.3 |
| SP_0662 | hk09, sensor histidine kinase | -2.2 |
| SP_0663 | Hypothetical protein | -2.0 |
| SP_0701 | pyrF, Orotidine 5’-phosphate decarboxylase | -2.2 |
| SP_0715 | lctO, lactate ocidase | -2.6 |
| SP_0717 | thiM, hydroxyethylthiazole kinase | -3.1 |
| SP_0726 | thiD Phosphomethylpyrimidine kinase | -3.3 |
| SP_0730 | spxB, pyruvate oxidase | -2.7 |
| SP_0766 | Sod, superoxide dismutase, manganese dependant | -2.3 |
| SP_0828 | rpiA, ribose-5- phosphate isomerase A | -2.2 |
| SP_0867 | Abc transporter, ABC binding protein | -2.7 |
| SP_0868 | Hypothetical protein | -2.9 |
| SP_0869 | Aminotransferase | -2.5 |
| SP_0875 | lacR, lactose phosohotransferase system repressor | -5.5 |
| SP_0876 | fruB, phosphofructokinase | -5.0 |
| SP_0877 | fruA, PTS system fructose specific component | -3.7 |
| SP_0958 | Hypothetical protein | -2.6 |
| SP_1127 | Hypothetical protein | -2.2 |
| SP_1215 | FNT family protein | -3.0 |
| SP_1227 | rr02, DNA binding response regulator | -2.1 |
| SP_1228 | A/G specific adenine glycosylase | -2.8 |
| SP_1276 | carA, carbamoyl phosphate synthase small subunit | -2.1 |
| SP_1289 | Hypothetical protein | -2.0 |
| SP_1415 | nagB glucosamine-6-phosphate isomerase | -2.1 |
| SP_1546 | Hypothetical protein | -2.1 |
| SP_1572 | dpr Starved cell/ iron storage peroxide induced protein | -2.5 |
| SP_1580 | msmK | -2.2 |
| SP_1587 | Oxalate formate antiporter | -4.9 |
| SP_1651 | Tpx, thiol peroxidase | -2.7 |
| SP_1683 | Sugar ABC transporter, sugar binding unit | -2.2 |
| SP_1684 | PTS-EII, IIBC component | -2.4 |
| SP_1685 | nanE | -2.7 |
| SP_1778 | Aquaporin | -2.4 |
| SP_1852 | galT, Galactose-1-phosphate uridylyltransferase, galactose metabolism | -2.5 |
| SP_1853 | galK, Galactokinase | -2.9 |
| SP_1861 | ProV, Choline transporter | -2.3 |
| SP_1862 | Hypothetical protein | -2.8 |
| SP_1863 | MarR, MarR family transcriptional regulator | -3.2 |
| SP_1883 | dexS, dextran glucosidase | -3.8 |
| SP_1884 | treP, Trehalosse PTS system | -3.7 |
| SP_1893 | Hypothetical protein | -2.2 |
| SP_1894 | gtfA, Sucrose phosphorylase | -4.9 |
| SP_1895 | msmG, Sugar ABC transporter, permease protein | -5.0 |
| SP_1896 | msmF, Sugar ABC transporter, permease protein | -4.7 |
| SP_1897 | msmE, Sugar ABC transporter, sugar binding protein | -5.5 |
| SP_1898 | aga, Alpha-galactosidase | -5.9 |
| SP_1996 | Universal stress protein | -2.2 |
| SP_2026 | Bifunctional acetaldehyde- CoA alcohol | -2.1 |
| SP_2054 | Hypothetical protein | -6.3 |
| SP_2107 | malM, 4-alpha-gluconotransferase | -2.0 |
| SP_2108 | Maltose/ maltodextrin ABC transporter | -2.3 |
| SP_2109 | Maltodextrin ABC transporter, permease protein | -2.3 |
| SP_2182 | Hypothetical protein | -2.8 |
| SP_2187 | Hypothetical protein | -5.0 |
| SP_2239 | htrA, Serine protease. | -2.2 |
| SP_rrnaB16S | Ribosomal RNA | -2.3 |
| SP_rrnaB23S | Ribosomal RNA | -3.5 |
| SP_rrnaC16S | Ribosomal RNA | -2.4 |
| SP_rrnaC23S | Ribosomal RNA | -3.4 |
| SP_rrnaD16S | Ribosomal RNA | -2.4 |
| SP_rrnaD23S | Ribosomal RNA | -3.4 |
